# Integrative genetic, genomic and transcriptomic analysis of heat shock protein and nuclear hormone receptor gene associations with spontaneous preterm birth

**DOI:** 10.1038/s41598-021-96374-9

**Published:** 2021-08-24

**Authors:** Johanna M. Huusko, Heli Tiensuu, Antti M. Haapalainen, Anu Pasanen, Pinja Tissarinen, Minna K. Karjalainen, Ge Zhang, Kaare Christensen, Kelli K. Ryckman, Bo Jacobsson, Jeffrey C. Murray, Stephen F. Kingsmore, Mikko Hallman, Louis J. Muglia, Mika Rämet

**Affiliations:** 1 PEDEGO Research Unit and Medical Research Center Oulu, University of Oulu, Oulu, Finland; 2 Department of Children and Adolescents, Oulu University Hospital, Oulu, Finland; 3 Division of Human Genetics, Center for Prevention of Preterm Birth, Perinatal Institute, Cincinnati Children’s Hospital Medical Center, Department of Pediatrics, University of Cincinnati College of Medicine, March of Dimes Prematurity Research Center Ohio Collaborative, Cincinnati, OH USA; 4 Institute of Public Health, University of Southern Denmark, Odense, Denmark; 5 Department of Epidemiology, College of Public Health and Department of Pediatrics, Carver College of Medicine, University of Iowa, Iowa City, Iowa, USA; 6 Department of Obstetrics and Gynecology, Sahlgrenska Academy, University of Gothenburg, Gothenburg, Sweden; 7 Department of Genetics and Bioinformatics, Area of Health Data and Digitalisation, Norwegian Institute of Public Health, Oslo, Norway; 8 Department of Pediatrics, University of Iowa, Iowa City, Iowa, USA; 9 Rady Children’s Institute for Genomic Medicine, Rady Children’s Hospital, San Diego, CA USA; 10 Burroughs Wellcome Fund, Research Triangle Park, NC USA; 11 Faculty of Medicine and Health Technology, Tampere University, Tampere, Finland

**Keywords:** Genetics, Medical research

## Abstract

Heat shock proteins are involved in the response to stress including activation of the immune response. Elevated circulating heat shock proteins are associated with spontaneous preterm birth (SPTB). Intracellular heat shock proteins act as multifunctional molecular chaperones that regulate activity of nuclear hormone receptors. Since SPTB has a significant genetic predisposition, our objective was to identify genetic and transcriptomic evidence of heat shock proteins and nuclear hormone receptors that may affect risk for SPTB. We investigated all 97 genes encoding members of the heat shock protein families and all 49 genes encoding nuclear hormone receptors for their potential role in SPTB susceptibility. We used multiple genetic and genomic datasets including genome-wide association studies (GWASs), whole-exome sequencing (WES), and placental transcriptomics to identify SPTB predisposing factors from the mother, infant, and placenta. There were multiple associations of heat shock protein and nuclear hormone receptor genes with SPTB. Several orthogonal datasets supported roles for *SEC63*, *HSPA1L*, *SACS*, *RORA*, and *AR* in susceptibility to SPTB. We propose that suppression of specific heat shock proteins promotes maintenance of pregnancy, whereas activation of specific heat shock protein mediated signaling may disturb maternal–fetal tolerance and promote labor.

## Introduction

Heat shock proteins (HSPs) are evolutionarily highly conserved and present in all cell types in all organisms. They constitute a large family of proteins that are classified according to approximate molecular weights ranging from 10 to 100 kDa: HSP10 (i.e., approximately 10 kDa HSPs), HSP40, HSP60, HSP70, HSP90, and HSP110. Some HSPs are expressed constitutively under normal conditions, whereas others are stress-induced under adverse environmental conditions such as heat, hypoxia, oxidative stress, infection, and inflammation^1–3^. The role of HSPs depends on their localization. Intracellular HSPs act as molecular chaperones and, together with co-chaperones, contribute to the maintenance of cellular homeostasis. Intracellular HSPs stabilize proteins against aggregation, mediate folding of newly translated proteins, and assist with protein translocation across intracellular membranes^1,4^. Extracellular or circulating HSPs are involved in activation of innate and adaptive immune responses^2,3^. It is known that infection and inflammation are significant risk factors in preterm birth^5^. Moreover, HSPs have a role in maturation and inactivation of nuclear hormone receptors (NRs) such as glucocorticoid, androgen, estrogen, and progesterone receptors^6,7^.

The incidence of preterm birth (i.e., birth before 37 completed weeks of gestation) varies from about 5% in Scandinavian countries^8^ to up to 19% in Bangladesh^9^. In approximately 70% of preterm deliveries, labor starts spontaneously. There are no effective ways to either predict or prevent spontaneous preterm birth (SPTB). One reason for this is limited knowledge of the pathways that regulate the timing of birth. The mechanisms leading to onset of normal term delivery likely comprise a complex interplay among fetus, placenta, and mother. In SPTB, it is thought that several pathological processes affect one or more labor-initiating factors^10^. Addressing single risk factors independently does not prevent preterm birth, suggesting that multiple etiologies are part of a complex parturition-initiating mechanism^11,12^.

HSPs are an important part of the developmental program and are among the first proteins expressed by the zygote after fertilization^13,14^. HSPs are also expressed during early pregnancy in both the embryo and maternal side of the placenta (i.e. decidua). Moreover, HSPs are expressed during neurulation, organogenesis, and on throughout fetal maturation^13,15^. In addition, formation of extra-embryonic tissue and organs (i.e. placenta) requires controlled temporal and spatial patterns of *HSP* expression^16^. HSP27, HSP60, HSP70, and HSP90, at least, are expressed in normal human placenta, and these HSPs have been suggested to play a role in cell viability and function^17^. On the other hand, abnormal HSP levels have been associated with pregnancy complications like transient hypertension, preeclampsia, preterm prelabor rupture of membranes (PPROM), and SPTB^18,19,20^.

Maternal and fetal genomes are estimated to contribute to the variation in timing of birth by 25–40%^21^. Several large studies investigating the genetic background of SPTB have been conducted^12,21,22^, several maternal loci have been robustly associated with preterm birth^23^, as has at least one fetal locus^24^. A previous study identified rare, likely damaging genetic variants of *HSPA1L* (HSP70 family) in Finnish mothers from families with recurrent SPTB^25^. Furthermore, *HSPA1L* showed an association with SPTB in a large genome-wide association study (GWAS) in a European American population^25^.

NRs have also been associated with SPTB. A recent study identified the glucocorticoid receptor (GR) signaling pathway as a candidate for SPTB risk^25^. The GR signaling pathway has crucial roles in glucose metabolism, growth development, and immune function, and may interact with progesterone, a key hormone required for normal pregnancy^26,27^.

Because HSPs have many roles during pregnancy and some have been linked to pregnancy complications including SPTB^25^, we sought to evaluate the importance of HSP coding genes, as well as HSP-regulated NRs, in relation to SPTB. We used multiple available data sources, such as GWAS, whole-exome sequencing (WES), and placental transcriptomic data to mine for evidence of HSP and NR gene involvement in susceptibility to SPTB.

## Results

HSPs have been linked to preterm birth^25^ and other pregnancy complications^18,28^. We investigated all 97 genes encoding members of the HSP families and all 49 genes encoding NRs, the targets of HSPs, for their role in SPTB susceptibility (Table S1 and S2, respectively). They included all HSPs and NRs in the UniProt database^29^ at the time of analysis. Pathway analysis showed HSPs and NRs were the most enriched in “Protein processing in endoplasmic reticulum” and “Estrogen signaling” KEGG pathways. Since estrogen is critical for maintenance of pregnancy and initiation of labor^30^, a role for HSPs and NRs in pregnancy and labor is plausible.

**Figure 1:**
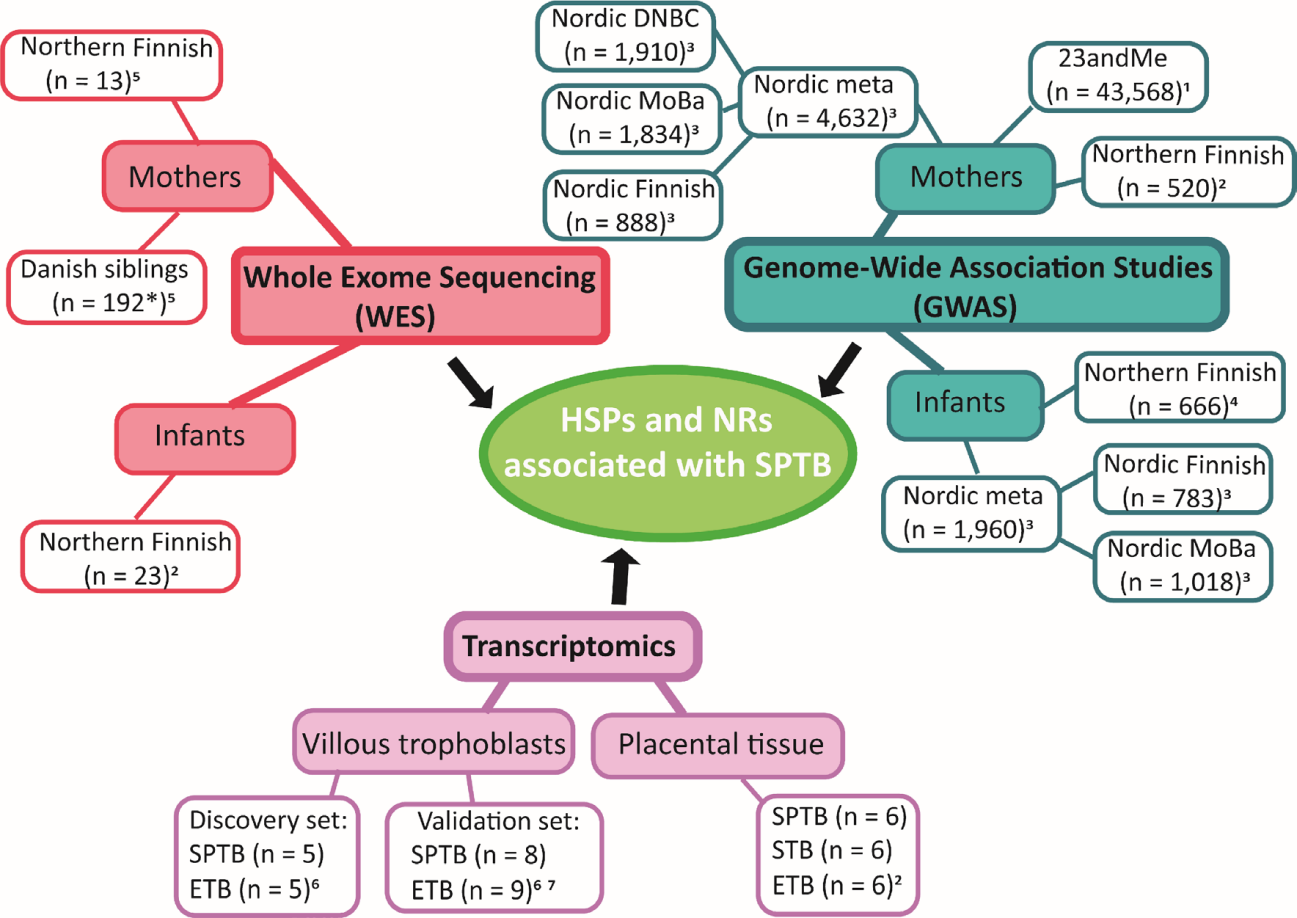
Summary of the datasets used in datamining. A genome-wide association study (GWAS) datamining included five different datasets [23andMe; Nordic metadata with Finnish, Danish and The Norwegian Mother, Father and Child Cohort Study (MoBa) subsets; and Northern Finnish dataset]. Whole exome sequencing (WES) was from two different datasets; the Northern Finnish and Danish population sets. Datamining of two transcriptomics datasets was conducted. Samples of the transcriptomics were from placentas from spontaneous preterm deliveries (SPTB) and spontaneous (STB) and elective (ETB) term controls. * Danish WES; n = 93 sister pairs and two families with three affected siblings. ^1^Zhang G et al. (2017) Genetic Associations with Gestational Duration and Spontaneous Preterm Birth^23^. ^2^Unpublished data. ^3^Zhang G et al. (2015) Assessing the Causal Relationship of Maternal Height on Birth Size and Gestational Age at Birth: A Mendelian Randomization Analysis^31^. ^4^Tiensuu H et al. (2019) Risk of spontaneous preterm birth and fetal growth associates with fetal SLIT2^32^. ^5^Huusko JM et al. (2018) Whole exome sequencing reveals HSPA1L as a genetic risk factor for spontaneous preterm birth^25^. ^6^Ackerman WE et al. (2016) Comprehensive RNA profiling of villous trophoblast and decidua basalis in pregnancies complicated by preterm birth following intra-amniotic infection^33^. ^7^Brockway HM et al. (2019) Unique transcriptomic landscapes identified in idiopathic spontaneous and infection related preterm births compared to normal term births^34^.

**Figure 2:**
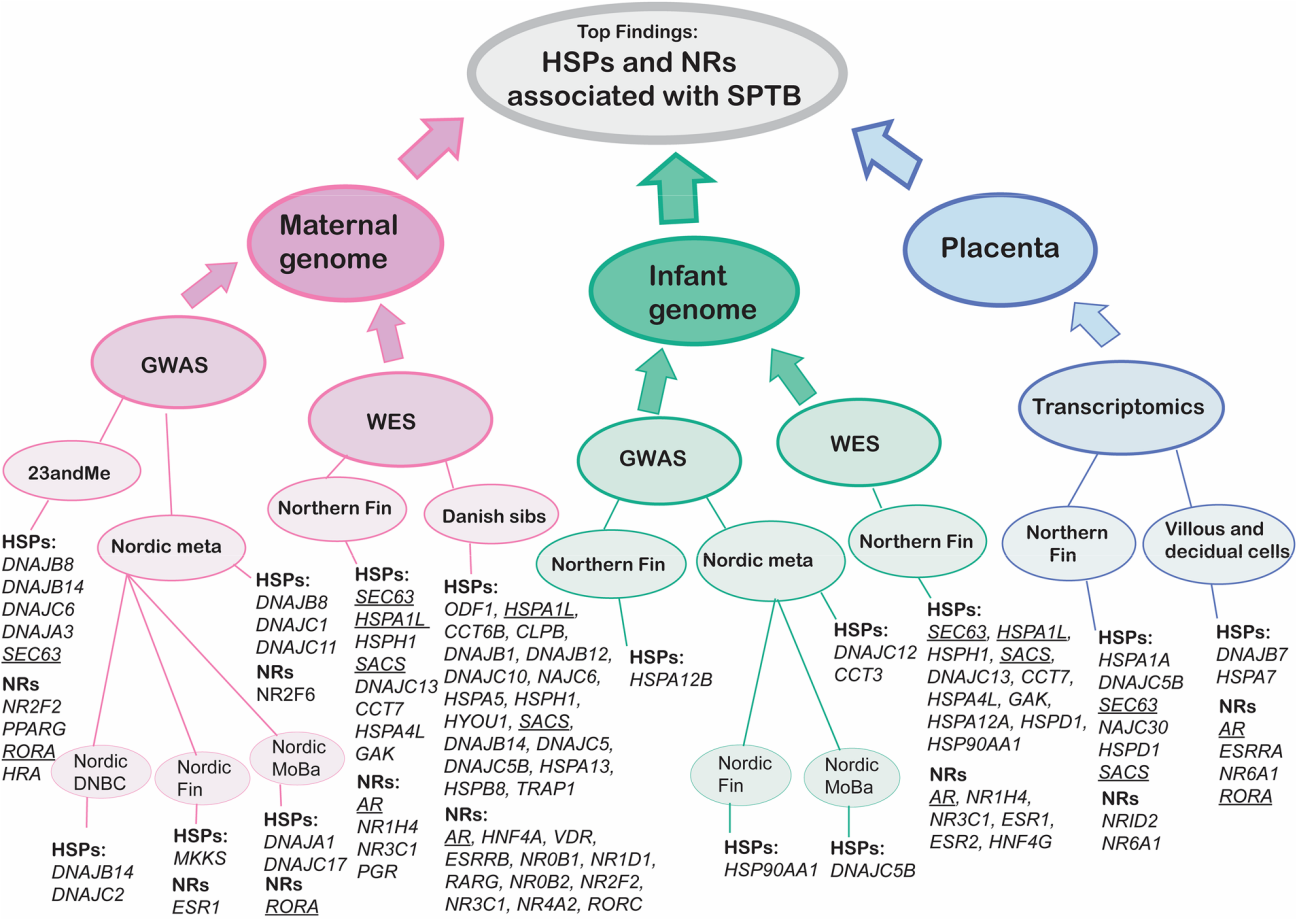
Top findings of HSPs and NRs associated with SPTB. Several datasets supported roles for *SEC63*, *HSPA1L*, *SACS*, *RORA*, and *AR* in susceptibility to SPTB. These genes are underlined in the figure.

A broad array of SPTB datasets, including GWAS, WES, and placental transcriptomics data, were screened for evidence suggesting associations with HSP and NR family members (Fig. 1). We first queried five GWAS datasets [23andMe (mothers only), Nordic metadata with Finnish, Danish, and Norwegian subsets, and a Northern Finnish dataset] to examine common variant associations using both “mother as affected” (giving birth preterm) and “child as affected” (born preterm). Secondly, we examined maternal and fetal exomes belonging to the Northern Finnish population set and in maternal exomes belonging to the Danish population set for potentially damaging, rare variants in HSP and NR genes. Thirdly, we sought changes in transcription of HSP genes in placentas from premature deliveries and spontaneous (STB) and elective (ETB) term controls. Findings are listed in Table 1, and main results are shown in Fig. 2.

**Table 1:** Combined results of GWAS, WES and transcriptome datasets.

	Method	Dataset name	Inclusion criteria	Heat shock protein genes	Nuclear Receptor Genes
**Maternal genome**	GWAS	23andMe	*p* < 1 × 10^−4^	*DNAJB8, DNAJB14, DNAJC6, DNAJA3, SEC63*	*NR2F2, PPARG, RORA, THRA,*
		Nordic Meta	*p* < 1 × 10^−4^	*DNAJB8, DNAJC1, DNAJC11*	*NR2F6*
		Nordic DNBC	*p* < 1 × 10^−4^	*DNAJB14, DNAJC2*	
		Nordic Fin	*p* < 1 × 10^−4^	*MKKS*	*ESR1*
		Norwegian Mother, Father and Child Cohort (MoBa)	*p* < 1 × 10^−4^	*DNAJA1, DNAJC17*	*RORA*
	WES	Northern Fin	MAF <10% and ACMG cat 1–3	*SEC63, HSPA1L, HSPH1, SACS,DNAJC13, CCT7, HSPA4L, GAK*	*AR, NR1H4, NR3C1, NR1D2, PGR*
		Danish sibs	MAF < 1%	*ODF1, HSPA1L, CCT6B, CLPB, DNAJB1, DNAJB12, DNAJC10, DNAJC6, HSPA5, HSPH1, HYOU1, SACS, DNAJB14, DNAJC5, DNAJC5B, HSPA13, HSPB8, TRAP1*	*AR, HNF4A, VDR, ESRRB, NR0B1, NR1D1, RARG, NR0B2, NR2F2, NR3C1, NR4A2, RORC*
**Infant genome**	GWAS	Norwegian Mother, Father and Child Cohort (MoBa)	*p* < 1 × 10^−4^	*DNAJC5B*	
		Nordic Fin	*p* < 1 × 10^−4^	*HSP90AA1*	
		Nordic meta	*p* < 1 × 10^−4^	*DNAJC12, CCT3*	
		Northern Fin	*p* < 1 × 10^−4^	*HSPA12B*	
	WES	Northern Fin	MAF <10% and cat 1-3	*SEC63, HSPA1L, HSPH1, SACS, DNAJC13, CCT7, HSPA4L, GAK, HSPA12A, HSP90AA1, CLPB, DNAJB13, DNAJC18, HSPD1*	*AR, NR1H4, NR3C1, ESR1, NR1H2, ESR2, HNF4G, VDR*
**Placenta**	Transcript-omic	Northern Fin	*p* < 0.05	*HSPA1A, HSPA1B, HSPA8, HSPA4L, DNAJB13, DNAJC5B, DNAJC11, DNAJC12, SEC63, DNAJC27, SACS, DNAJC30, HSP90AB1, HSP90B1, HSPD1, BBS10*	*NR113, NRID2, RARB, NR4A3, NR6A1, NXRA, NR2F6*
		Placental villous and decidual cells	*p* < 0.05	*DNAJB7, HSPA7*	*AR, ESRRA, NR6A1, RORA*

Genes implicated in orthogonal datasets are underlined.

### Several HSP and NR genes have suggestive association signals in GWAS datasets

In the five GWAS datasets we sought significant (*p* < 5 × 10^−8^) and suggestive (*p* < 1 × 10^−5^) associations between SPTB and 100-kb windows surrounding each of 97 HSP and 49 NR genes. For comparing HSP and NR variants with exome data and transcriptomics, we extracted all GWAS loci with *p* < 1 × 10^−4^ to construct a large preliminary gene set of potential importance in SPTB. The GWAS datasets supported roles for multiple HSP and NR genes in SPTB susceptibility (Fig. 2, Table 1).

Five HSP genes (*DNAJB8*, *DNAJB14*, *DNAJC6*, *DNAJA3*, and *SEC63*) exhibited potentially significant associations with mothers giving birth preterm (*p* < 4 × 10^−9^–1 × 10^−4^) in the 23andMe dataset (Table S3). A previously reported, genome-wide significant signal (*p* < 1 × 10^−8^) was detected upstream of *DNAJB8* in an intron of *EEFSEC*, and the association replicated in the Nordic dataset (*p* < 1 × 10^−4^) (Table S4)^23^. *DNAJB14* had a suggestive association (*p* < 1 × 10^−5^) that replicated in the maternal Nordic Danish data (*p* < 1 × 10^−4^, Tables S3 and S4). Six other genes (*DNAJA1*, *DNAJC1*, *DNAJC2*, *DNAJC11*, *DNAJC17*, and *MKKS*) were detected at the alpha level of *p* < 1 × 10^−4^ in the maternal Nordic datasets (Table S4). Most of the maternal HSP SNP associations in the Nordic data overlapped with those in the 23andMe data.

Among NR genes, suggestive maternal associations (*p* < 1 × 10^−5^) with SPTB were observed in *NR2F2* and *THRA*, and *PPARG* and *RORA* had variants with *p* < 1 × 10^−4^ (Table S5, 23andMe dataset). In the Nordic datasets, variants in *RORA*, *ESR1*, and *NR2F6* had *p* values < 1 × 10^−4^ (Table S6). No associations were seen in the Northern Finnish maternal data.

In the GWAS datasets in which the infant was treated as affected, only one variant in *HSPA12B* showed a suggestive association (*p* < 1 × 10^−5^) in the Northern Finnish data. Variants in *HSP90AA1*, *DNAJC5B*, *DNAJC12*, and *CCT3* had *p* values of < 1 × 10^−4^ in the Nordic Finnish, MoBa, or meta-analysis datasets (Tables S7 and S8). In the maternal GWAS data, significant and suggestive association signals were detected for *DNAJB8*/*EEFSEC* and *DNAJB14*, respectively; these associations were replicated in independent GWAS datasets. In addition, many variants with *p* < 1 × 10^−4^ were shared across different GWASs in both maternal and fetal data. After GWAS, we investigated the presence of potentially damaging variants in the HSP and NR genes from maternal and fetal exomes.

### Potentially damaging variants of HSP and NR genes identified in whole exome sequence

The presence of rare [minor allele frequency (MAF) < 1%] and common (MAF 1–10%), potentially damaging variants [category 1–3 in accordance with classification of the American College of Medical Genetics (ACMG)^35^] in the HSP and NR genes was investigated in maternal and fetal exomes. Among affected Northern Finnish individuals (either giving birth preterm or born preterm), we found 15 HSP genes and ten NR genes with potentially damaging heterozygous variants in multiple individuals or single individuals with multiple, potentially damaging variants in a single gene. We found 18 HSP and 12 NR genes with rare, possibly damaging variants that were shared by affected Danish maternal sibling pairs. Genes *CCT7*, *HSPA1L*, *HSPA5*, *HYOU1*, *SACS*, *SEC63*, *AR,* and *NR1H4* had rare, potentially damaging variants in both Finnish and Danish affected exomes. We previously reported the presence of rare, damaging variants in Heat Shock Protein 70-kDa-like 1 (*HSPA1L*) in Finnish and Danish families with recurrent SPTBs^25^. One of the variants, rs34620296, also showed a trend toward significance in the 23andMe GWAS data (*p* = 1 × 10^−3^; MAF in cases, 0.0025 and MAF in controls, 0.0010), and was previously shown to reduce chaperone activity^36^ and affect decidualization^25^.

Sacsin (*SACS*), a HSP gene associated with spastic ataxia (OMIM: #270,550)^37,38^, had four potentially damaging (ACMG category 1–3) missense variants in the Finnish exomes (rs192610957, rs144267558, rs116907814, and rs17325713) and two missense variants in the Danish exomes (rs147099630 and chr13:23915410G > A, p.H119Y). According to the Combined Annotation Dependent Depletion (CADD, v1.6)^39^ score (> 30), these variants are among the top 1% of deleterious variants in the human genome. Rs192610957 and rs116907814 were enriched in the Finnish population compared to the general European population (MAF 0.007 *vs*. 0.0005, and 0.011 *vs*. 0.0009, respectively) (http://www.sisuproject.fi/; https://gnomad.broadinstitute.org/).

In both Finnish and Danish families, *HSPA5* variant rs56136100 was shared by two affected mothers within a family. Rs56136100 is a non-conservative, missense variant (p.Glu557Gly) that is predicted to be damaging by multiple in silico tools (SIFT v6.2.1^40^, PolyPhen-2 v2^41^, and MutationTaster v2^42^) and has a CADD v1.6 score of 33. This missense variant, could potentially affect the physiochemical properties of HSPA5*,* as the sequence variant causes a change from an acidic (Glu) to a hydrophobic (Gly) amino acid.

The androgen (dihydrotestosterone) receptor, *AR*, a NR gene associated with androgen insensitivity syndrome (OMIM: #300,068), harbored five potentially damaging variants: rs137852593 and rs5031002 (Finnish exomes), and rs201934623; chrX:66766114C > A, p.P186T; and chrX:66900678A > G, p.R628G (Danish exomes). In addition, several individuals who belonged to the Finnish families had longer *androgen receptor* (*AR*) exon-1 CAG_n_ repeats, together with *HSPA1L* rs34620296, which was previously associated with SPTB^25^. Furthermore, in our previous study, longer *AR* CAG_n_ repeats were overrepresented in preterm infants compared to term controls^43^.

In the progesterone receptor (*PGR*), two variants, rs11571145 and rs11571222, were shared by Northern Finnish affected mothers in two different families. According to RegulomeDB (www.regulomedb.org), rs11571145 (p.Pro186Leu in the PGR-B isoform) is annotated as likely to affect binding to DNA. This variant is also predicted to be damaging and disease causing by the in silico tools SIFT v6.2.1^40^ and MutationTaster v2^42^. Moreover, in the family with rs11571145, two affected mothers also shared common missense variants rs1042838 and rs3740753, which showed nominal significance (*p* = 0.007 and *p* = 0.008, respectively) for gestational age (GA) in the 23andMe data as well as a trend (*p* = 0.074, effect (eff) =  − 1.213 and *p* = 0.046, eff =  − 1.362, respectively) in the maternal Nordic metadataset. Variants rs11571145, rs1042838, and rs3740753 are missense variants (Pro22Leu, Val496Leu, and Ser180Thr, respectively, in the PGR-A isoform, and Pro186Leu, Val660Leu, and Ser344Thr, respectively, in the PGR-B isoform). The role of different PGR isoforms in pregnancy is well supported^44,45^. Especially, the expression of low affinity variant of PGR, PGR-A increases towards term labor^46^.

The WES datasets also supported roles for HSP and NR genes in SPTB susceptibility (Fig. 2, Table 1). Genes *CCT7*, *HSPA1L*, *HSPA5*, *HYOU1*, *SACS*, *SEC63*, *AR,* and *NR1H4* had rare, potentially damaging variants in both Finnish and Danish exomes. Both genetic variation in HSP genes and differences in their expression are associated with SPTB^18,19,20^. Thus, after datamining the genetic datasets, we investigated changes in HSP and NR mRNA expression in the placenta by investigating transcriptomics datasets.

### Placental transcriptomics identify differences in HSP and NR gene expression during SPTB

Expression levels of HSPs change during pregnancy, especially in complicated pregnancies^28^, suggesting that changes in HSP expression might have a role in pregnancy complications. To investigate whether HSP and NR expression levels change in SPTB, we examined RNA levels of 97 HSP genes and 49 NR genes in placentas from premature deliveries and term controls from Northern Finland. We compared expression levels in three groups: spontaneous preterm birth (SPTB, n = 6), spontaneous term birth (STB, n = 6), and elective term birth (ETB, n = 6). 15 HSP genes (Table 2) and seven NR genes (Table 3) were significantly up- or downregulated (*p* < 0.05) in groupwise comparisons.

**Table 2:** Significant (*p* < 0.05) differences in HSP gene regulation among SPTB, STB, and ETB in placental transcriptomics data.

Gene	SPTB vs. STB		SPTB vs. ETB	
	Fold change	*p* Value^1^	Fold change	*p* Value^1^
*HSPA1A*	1.95	0.012	2.27	0.004
*HSPA1B*			2.09	0.011
*HSPA8*			1.25	0.041
*HSPA4L*			3.88	0.004
*DNAJB13*			− 4.43	0.010
*DNAJC5B*	− 2.86	0.017		
*DNAJC12*			3.89	0.031
*SEC63*	− 1.18	0.045		
*DNAJC27*			1.43	0.015
*SACS*			1.75	0.038
*DNAJC30*	− 1.20	0.042	− 1.21	0.042
*HSP90AB1*			1.18	0.013
*HSP90B1*			− 1.23	0.025
*HSPD1*	1.27	0.002	1.35	0.00027
*BBS10*			1.29	0.032

^1^Nominal association.

**Table 3:** Significant (*p* < 0.05) differences in NR gene regulation among SPTB, STB, and ETB in placental transcriptomics data.

Gene	SPTB vs. STB		SPTB vs. ETB	
	Fold change	*p* Value^1^	Fold change	*p* Value^1^
*NR1I3*			3.25	0.001
*NR1D2*	− 1.39	0.049		
*RARB*			1.73	0.027
*NR4A3*			4.30	0.037
*NR6A1*	2.38	0.006	2.27	0.009
*RXRA*			− 1.26	0.027
*NR2F6*			− 1.31	0.035

^1^Nominal association.

These results imply that expression levels of multiple HSP and NR genes change in preterm birth. The most robust changes in placental gene expression were *HSPA1, DNAJC30, HSPD1,* and *NR6A1,* which exhibited congruent, significant (*p* < 0.05) differences in comparisons of SPTB vs. spontaneous term placentas and SPTB vs. elective term placentas. This suggests that mRNA expression changes in these genes are associated with prematurity, rather than spontaneous labor.

### Top genes in placental villous and decidual cells from discovery and validation SPTB vs. elective term birth datasets

We also compared RNA expression levels in maternal placental tissues (decidua basalis) and fetal placental tissues (villous tissue) in spontaneous preterm births (SPTB, n = 5) and elective term births (ETB, n = 5)^33,34^. *DNAJB7*, *AR*, and *ESRRA* were upregulated in SPTB vs. ETB in the decidua, whereas *HSPA7*, *NR6A1* and *RORA* were downregulated in SPTB compared to ETB in villous tissue (Table 4, *p* < 0.05, *t *test).

**Table 4:** Differentially regulated SPTB vs. ETB transcripts in the discovery set.

**Tissue**	**Gene group**	**Gene**	**Fold change**	***p* Value**
**UPREGULATED GENES**				
**Decidua basalis**	HSP	*DNAJB7*	1.75	0.0473
	NR	*AR*	1.82	0.0158
	NR	*ESRRA*	1.50	0.0282
**DOWNREGULATED GENES**				
**Tissue**	**Gene group**	**Gene**	**Fold change**	***p V*alue**
**Villous tissue**	HSP	*HSPA7*	1.51	0.0480
	NR	*NR6A1*	1.29	0.0112
	NR	*RORA*	1.44	0.0342

Some of these findings were mirrored in the validation datasets. *NR6A1* was among the top findings in placental transcriptomics data that originated in northern Finland, whereas modest association signals (*p* < 0.0001) were detected for the region encompassing *RORA* in 23andMe and Nordic GWAS datasets.

### Confirmation of SPTB associations in orthogonal datasets

We datamined multiple GWAS, WES, and transcriptomics datasets from mothers, infants, and placenta. HSPs and NRs that are potentially associated with SPTB were found in all these datasets. Multiple HSPs and NRs (Table 1, Fig. 2) have potential roles in SPTB susceptibility in at least one of the datasets. Finally, we compared the results from different analyses. Several datasets supported roles for *SEC63*, *HSPA1L*, *SACS*, *RORA*, and *AR* associations with spontaneous preterm birth (Table 1, Fig. 2).

## Discussion

Previous studies have suggested a role for certain HSPs in pregnancy complications, which led us to look for HSPs and NRs that might affect SPTB risk. Analysis of GWAS, WES, and transcriptomics data from mothers, infants, and placentas, revealed HSP and NR gene associations with SPTB in each (Table 1). More significantly, we identified several HSPs and NRs with associations with SPTB in multiple, orthogonal datasets – notably *SEC63*, *SACS*, *RORA*, *AR,* and *PGR*. Previous studies have suggested that SPTB to be attributable to multiple pathological processes^11^. Thus, varying pathways leading to SPTB could partly explain the variations in the results among the datasets. Maternal, and also to some extent fetal, genomes affect the susceptibility to preterm birth and duration of pregnancy in general^23,47,48^. We analyzed several maternal and fetal GWAS and WES datasets to identify HSP and NR genes associated with preterm birth. *DNAJB8, DNAJB14, SEC63,* and *RORA* showed associations in at least two genomic datasets.

Previous studies have indicated that in addition to changes in levels of HSP expression, changes in the distribution/relative concentration of different HSPs could lead to pregnancy complications. For example, early changes in the ratio of circulating HSP60 to HSP70 have been shown to predict miscarriage^14^. Additionally, there are well-defined temporal and spatial patterns of HSP expression in the human placenta^13^. Changes in HSP expression could affect placental pathology and cause pregnancy complications like preterm birth. Consequently, we also searched for SPTB-associated changes in HSP mRNA expression in the placenta. A comparison of SPTB and placentas from spontaneous and elective term pregnancies (Table 2) indicated that multiple HSPs differed in expression.

Elevated circulating HSP concentrations have previously been associated with increased risk of pregnancy complications such as preeclampsia and preterm delivery^3,28,49^. Circulating HSPA1A (of Hsp70 family) levels are elevated in patients at high risk for preterm delivery^50^. In our current study, *HSPA1A* mRNA expression was upregulated in SPTB placentas compared to placentas from term pregnancies. Circulating HSPA1A levels are downregulated in women with a normal pregnancy compared to in nonpregnant women^28,50,51^. Extracellular HSPA1A may be removed by innate immune mechanisms as part of tolerogenic changes in the immune system and, as a result, may promote the maintenance of immunological tolerance to the fetus. The ability of extracellular HSPA1A to elicit immune responses might be harmful in pregnancy and could lead to maternal immune rejection of the fetus^28^. By disturbing this tolerance, upregulation of *HSPA1A* during pregnancy could increase the risk of preterm labor. In contrast to HSPA1A, some HSPs, like Hsp60, are present in the peripheral circulation of healthy nonpregnant and pregnant individuals^52,53^. Protein levels of some HSPs increase along with advancing gestational age, which may reflect their involvement in initiation of labor^54^. Suppression of HSP production during pregnancy could be an important mechanism for maintaining pregnancy. However, it is also possible that elevated HSP levels are a consequence of harmful conditions such as preterm labor and a sign of the body’s attempt to maintain homeostasis.

HSPs are essential to the maturation and inactivation of NRs. In this study, we found rare, potentially damaging variants located within the exons of PGR and AR receptor genes from families with multiple SPTB. Moreover, in 23andMe and Nordic GWAS datasets *RORA* associated with SPTB. Progesterone has an essential role in the maintenance of pregnancy^55^. Progesterone withdrawal has been noted to result in parturition in some animals, but plasma levels of progesterone in humans remain high until the placenta is removed. On the other hand, PGRs are potential regulators of timing of birth^56^. Progesterone and PGRs may have a role in anti-inflammatory responses in the myometrium, and impaired function of PGRs may lead to initiation of labor. Moreover, AR has important roles during pregnancy^57^ and has previously been linked to pregnancy complications. For example, longer *AR* CAG_n_ repeats are overrepresented in women with recurrent spontaneous abortions^58^ and in SPTB infants^43^, and increased *AR* expression has been observed in the placentas of preeclamptic women^59^. Additionally, higher AR ligand levels result in myometrial contractions, cervical dilatation and in preterm birth^57^. RORA, on the other hand, regulates genes involved in inflammatory response and circadian rhythm, for instance^60–62^. Changes in circadian rhythm have been shown to associate with placental detachment and SPTB^61,62^.

According to a KEGG pathway analysis, many HSP and NR genes (*HSPA1A, HSPA1B, HSPA1L, HSPA2, HSPA6,* and *HSPA8*), including three HSP90 genes (*HSP90AA1*, *HSP90AB1*, and *HSP90B1*), play a role in the estrogen signaling pathway. Estrogen signaling is one of the main pregnancy-associated pathways in which HSPs and NRs play a role. In the absence of estrogenic ligands, estrogen receptor (ER), like other nuclear hormone receptors, is assembled into an Hsp90-based chaperone protein complex, which keeps the ER in a ligand binding–competent but inactive state. A total of 21 HSPs and three HSP co-chaperones have been found to associate with the ER^63^. Estrogen signaling is necessary for a successful pregnancy, as estrogen is required for processes such as proliferation of the myometrium before term and the contractile response that leads to parturition at term^64^. Thus, changes in HSP genes or protein expression could affect estrogen signaling and cause pregnancy complications, promoting preterm birth.

SEC63 (of Hsp40 family) was identified to associate with SPTB in GWAS, WES and placental transcriptomic data. SEC63 act as a co-chaperone, is a component of the protein translocation machinery in endoplasmic reticulum and associates with decidualization in early pregnancy^65,66^. The damaging variants of *SEC63* and lower expression levels of SEC63 in the placenta might affect implantation site and decidualization predisposing to SPTB. Another Hsp40 family member, *SACS* had damaging variants in both Finnish and Danish affected exomes and was upregulated in the placentas of SPTB. It has been suggested that SACS is a key player in cellular protein quality control system and in organizing proteins into bundles called intermediate filaments^67,68^. Being part of protein quality control, increased levels of SACS in the placenta could be due to a consequence of harmful conditions that are responsible for SPTB.

One of the important GWAS findings of the present study were in a region that has been shown to loop to *DNAJB8* [DnaJ Heat Shock Protein Family (Hsp40) Member B8].The looping region has a genome-wide significant signal associated with SPTB^23^. DNAJB8 has a role in suppressing aggregation and toxicity of polyglutamine proteins [i.e., proteins containing polyglutamine (polyQ) regions that are encoded by repetitive CAG or CAA DNA sequences]. Proteins with expanded polyQ regions can cause pathogenic phenotypes (e.g., neurodegenerative phenotypes), and intermediate levels of polyQ expansion can influence host cell susceptibility to misfolded pathogenic protein; however, other studies have proposed that polyQ aggregates can be benign or even offer protection from toxicity associated with smaller, oligomeric conformers^69^.

HSPs have important roles starting at the beginning of pregnancy, and they are among the first proteins expressed by the zygote after fertilization. They are expressed during early pregnancy stages in both the embryo and maternal decidua. For example, they maintain the integrity of intracellular proteins^14^. A recent study revealed that HSPs from placental mitochondria may be associated with trophoblast differentiation^70^. As HSPs have many important functions throughout pregnancy, it is plausible that changes in either HSP genes or their expression could compromise maintenance of normal pregnancy, leading to SPTB. On the other hand, HSPs are involved in activation of the innate and adaptive proinflammatory immune response. It is well established that infection and inflammation represent a highly significant risk factor in preterm birth^5^. Thus, it is possible that the changes in HSP expression associated with preterm birth are due to activation of inflammation-related pathways.

There were some clear limitations in our study. First, we could not differentiate whether a specific gene associates with early preterm or late preterm birth. In our analysis, the preterm data consisted of specimens obtained both before 30 weeks and after 30 weeks of gestation Second, there are likely differences how placental samples were collected. There were three different data sets utilizing tissue samples from human placenta. Two of these data sets were already published^33,34^. It may be that not all the placental samples have been collected according to standard as proposed by the International Federation of Placenta Associations. These methodological limitations decrease likelihood to detect gestational age specific associations and complicates comparisons between the data sets originating from placental tissues.

In conclusion, GWAS, WES, and transcriptome datasets indicated that various HSPs and NRs are associated with SPTB susceptibility. Further studies are required to resolve the exact roles of different HSPs and NRs in SPTB. The length of pregnancy is a species-specific which makes it challenging to study human pregnancy. However, for example, gene knock-down in relevant cell line could allow in vivo studies of gene function and identify specific pathways in preterm birth. We propose that activation of HSP signaling disturbs maternal–fetal tolerance and promotes susceptibility to early labor. Mechanistic studies are needed to resolve how genetic variants of *HSP* and *NR* genes compromise maintenance of normal homeostasis to support normal pregnancy or promote initiation of events leading to SPTB.

## Methods

### GWAS data

We used multiple available sources of preterm birth GWAS data, including both maternal and fetal genomes. The first dataset we used comprised 43,568 mothers of European ancestry; these mothers were identified from among 23andMe’s research participants as described previously^48^. The second dataset included meta-analysis data for 4632 mothers and their 1960 infants from three independent Nordic (Finnish, Danish, and Norwegian) preterm birth case/control data sets of European ancestry^31^. In this dataset, preterm samples were enriched, and samples from borderline preterm and early term (gestation age 37–38 weeks), as well as post-term (gestation age > 42 weeks), births were excluded. The third dataset included 608 mothers with spontaneous preterm (gestation age < 36 weeks) or term (gestation age 38–41 weeks) deliveries and their preterm or term born children. This dataset originated exclusively from northern Finland, and a full data description was presented previously in detail^32^.

All preterm births included in the Nordic or Northern Finnish population sets were spontaneous. Obstetrical induction of labor, placental abnormalities, preeclampsia, congenital malformations, and multiple births were excluded. Pregnancies involving preexisting medical conditions known to be associated with preterm birth and pregnancies with complications were also excluded.

### WES data

We had two population sets with WES data available. The first was a dataset of Northern Finnish mothers with preterm deliveries (*n* = 13)^25^ and their children (*n* = 23) who were born preterm (gestation age < 36 weeks). This population set comprised seven unrelated families with a strong family history of recurrent SPTBs; the set was selected retrospectively from the birth diaries of Oulu University Hospital from 1973 to 2003 and prospectively from 2003 to 2005. Selection criteria were described previously in detail^43,71^. Another population set of European ancestry with available WES data was from Denmark and included 192 women from 95 families: 93 affected sister pairs (both sisters had given birth preterm) and two sister triads with preterm deliveries occurring before 37 completed weeks of gestation. All women had experienced at least one PTB and, in the majority of the sister pairs (83%), both sisters had experienced a SPTB.

WES for both of the Finnish and Danish exomes was performed as previously described in detail^25^. In short, next-generation exome sequencing of the Finnish samples was performed at the Center for Pediatric Genomic Medicine, Children’s Mercy Hospital (CMH; Kansas City, MO, USA). Exome samples were prepared with the Illumina Nextera Rapid Capture Exome kit and sequenced with the Illumina HiSeq 2500 instrument. Sequence data were generated with Illumina RTA 1.18.64.0 and bcl2fastq-1.8.4 and aligned against the reference human genome (GRCh37.p5). Variant calls were made with the Genome Analysis Toolkit (GATK v4.2.0.0)^72^, and duplicate reads were identified and flagged with the Picard MarkDuplicates tool^72^. Exomes of the Danish sample set were sequenced with the Complete Genomics platform (BGI, Shenzhen, China) by using the manufacturer’s pipeline. Reads were aligned against the National Center for Biotechnology Information (NCBI) build 37 human reference genome.

For the Finnish exomes, we used variant annotation data from the Center for Pediatric Genomic Medicine’s CMH Variant Warehouse database (http://warehouse.cmh.edu), including frequency data for approximately 3900 individuals previously sequenced at the center^73^. Pathogenicity was categorized according to the ACMG^35^ as: 1, previously reported to be disease-causing; 2, expected to be pathogenic (loss of initiation, premature stop codon, disruption of stop codon, whole-gene deletion, frame shifting indel, and disruption of splicing); and 3, unknown significance but potentially disease-causing (nonsynonymous substitution, in-frame indel, disruption of polypyrimidine tract, and overlap with 5′ exonic, 5′ flank, or 3′ exonic splice contexts). Only variants that fit one of these criteria (1–3) were considered interesting. For the Danish exomes, we used Ingenuity Variant Analysis software (Qiagen) and included only rare (MAF < 1%) likely damaging variants that were shared by the affected sisters in each family.

### Placental transcriptomics data

We used available transcriptomics data to explore HSP and NR levels in human placenta. Placental tissues were collected at Oulu University Hospital in 2012–2014, and all samples were from uncomplicated preterm or term pregnancies as described previously^74^. Each placenta was inspected in terms of morphology; weight, size, cord position, infarcts and calcification were recorded. In short, samples were collected from the basal plate immediately underneath the placental surface (the maternal side of placenta). The transcriptomic set consisted of placenta samples resulting from spontaneous vaginal deliveries that occurred either preterm (SPTB; gestation age < 36 weeks, *n* = 6) or term (STB; gestation age > 38 weeks, *n* = 6), and from elective caesarean deliveries without signs or symptoms of labor at term (ETB; gestation age > 38 weeks, *n* = 6).

Transcriptomics data were generated from RNA isolated from the basal plate of placentas with the Qiagen Rneasy Micro kit. RNA quality was assessed with the Agilent RNA 6000 Nano kit in the Agilent 2100 Bioanalyzer instrument. The samples were sequenced with the HiSeq2500 instrument using paired-end sequencing chemistry with 100 bp read length. Number of lanes used in sequencing was 1. The reads obtained from the instrument were base called using the instrument manufacturer’s Bcl2fastq version 1.8.4 base calling software. Read quality droped at the ends of the reads and thus quality trimming was needed. Trimming of reads was done with Trimgalore version 0.3.3 (https://www.bioinformatics.babraham.ac.uk/projects/trim_galore/) and Cutadapt (v1.1)^75^. The reads were aligned against the human reference genome (hg19 assembly, downloaded from UCSC) using TopHat version 2.0.10.1 (https://ccb.jhu.edu/software/tophat/index.shtml). Only uniquely aligned reads were used for the further analysis.

Next the reads were associated with known genes based on RefSeq annotations derived from UCSC database and the number of reads associated with each gene was counted using HTSeq tool version 0.6.1 (https://htseq.readthedocs.io/en/master/). Here the counts were normalised using the TMM normalisation method of the edgeR R/Bioconductor package^76^. For statistical testing the data were further transformed using the voom approach in the limma package^77^.

Data were normalized to remove variations among the samples. Finally, there was a very high correlation among the samples. The thresholds used in filtering the differentially expressed genes were *p* values of < 0.05. The placental transcriptomics data were deposited in NCBI’s Gene Expression Omnibus (GEO) (https://www.ncbi.nlm.nih.gov/geo) and are accessible through GEO Series accession number GSE120480.

### RNA expression data from placental villous and decidual cells; discovery dataset

We used publicly available RNA sequencing data (GEO dataset ID: GSE73714)^33^ from paired villous trophoblast and decidua basalis specimens collected from spontaneous idiopathic preterm birth (SPTB; gestation age 30–33 weeks, *n* = 5) or term birth (by caesarean section, absence of labor; i.e., ETB; gestation age 38–39 weeks, *n* = 5). Details of the RNA sequencing and data generation were described previously^33^. In short, total RNA was extracted from flash-frozen specimens with TRIzol. RNA-seq libraries were constructed with the TruSeq Stranded Total RNA Sample Prep Kit with Ribo-Zero Gold (Illumina) and sequenced with the Illumina HiSeq 2500 platform. Illumina Analysis pipeline in HiSeq Control Software v2.2.38 was used for image analysis, base calling, and error estimation. Reads were mapped to UCSC hg38, and data were normalized by using Bioconductor statistical packages (https://www.bioconductor.org/).

### RNA expression data from villous trophoblasts; replication/metadataset

This dataset included placental villous samples collected from pregnancies that resulted in SPTB (gestation age 29–36 weeks, *n* = 8) or term (gestation age 38–42 weeks, *n* = 9) deliveries. Full data description, generation, and analysis were explained in detail elsewhere^34^. In short, total RNA was prepared from snap-frozen biobank placental samples and submitted to the University of Cincinnati Genomics, Epigenomics and Sequencing Core for RNA sequencing with the RiboZero kit (Illumina) and Illumina High Seq 2100 system for library preparation and sequencing, respectively. To increase statistical power, RNA sequence data obtained from these samples were combined with previously published placental villous transcriptomes (GEO GSE73714 term, *n* = 5 and SPTB, *n* = 5)^33^. We compared RNA expression levels (counts per million, CPM) between SPTB and term births and generated heatmaps with GraphPad Prism version 8.0 (https://www.graphpad.com/scientific-software/prism/).

### Ethics statement

All experiments were performed in accordance with relevant guidelines and regulations. Written informed consent was obtained from all of the individuals or their guardians who participated in this study. Study methods for the Northern Finnish WES, GWAS, and placental transcriptomics data were approved by the ethics committee of Oulu University Hospital (79/2003, 14/2010, and 73/2013), and for the Danish WES by the University of Southern Denmark (NVK#1302824) and the University of Iowa (IRB#200608748). Individuals in the large GWAS (European American women) were research participants of 23andMe, Inc., a personal genetics company. All of the 23andMe participants provided informed consent and participated in the research online, under a protocol approved by the external AAHRPP-accredited institutional review board Ethical & Independent Review Services (E&I Review). For the Nordic GWAS data, the study was approved by the ethics committees of Oulu University Hospital and Helsinki University Central Hospital (Finnish subset), as well as The Regional Committee for Medical Research Ethics in Norway, 7021/2010/2683 REK South-Eastern (The Norwegian Mother, Father and Child Cohort Study subset). Written informed consent was given by all participants. The study involving villous tissue specimens was approved by the Cincinnati Children’s Hospital Medical Center institutional review board (#IRB 2013-2243, 2015-8030, 2016-2033).

## Supplementary Information

Supplementary Information.
