# Supplementary material for: Integrative genetic, genomic and transcriptomic analysis of heat shock protein and nuclear hormone receptor gene associations with spontaneous preterm birth
Source: Sci Rep. 2021 Aug 24;11:17115. doi: 10.1038/s41598-021-96374-9 (PMC8384995; doi:10.1038/s41598-021-96374-9)
Supplement: Supplementary file 1 — Supplementary Information. [file 41598_2021_96374_MOESM1_ESM.docx]

**Supplementary Information**

**Integrative Genetic, Genomic and Transcriptomic Analysis of Heat Shock Protein and Nuclear Hormone Receptor Gene Associations with Spontaneous Preterm Birth**

**Short title:** HSP and NR variants associated with spontaneous preterm birth

Johanna M. Huusko^1,2^, Heli Tiensuu^1^, Antti M. Haapalainen^1^, Anu Pasanen^1^, Pinja Tissarinen^1^, Minna K. Karjalainen^1^, Ge Zhang^2^, Kaare Christensen^3^, Kelli K. Ryckman^4^, Bo Jacobsson^5^, Jeffrey C. Murray^6^, Stephen F. Kingsmore^7^, Mikko Hallman^1¶^, Louis J. Muglia^2,8¶*^, Mika Rämet^1,9¶*^

**Figures**

**Figure S1. Overview of the workflow.**

**Tables**

**Table S1. Heat shock protein (HSP) families and genes.**

**Table S2. Nuclear hormone receptor (NR) genes.**

**Table S3. Variants with *p* < 0.0001 in HSP gene regions +/- 100 kb in maternal 23andMe SPTB GWAS data.**

**Table S4. Variants with *p* < 0.0001 in HSP genes +/- 100 kb in the maternal Nordic SPTB GWAS data. Table S5. Variants with *p <* 0.0001 in NR genes in the maternal 23andMe SPTB GWAS data.**

**Table S6. Variants with *p <* 0.0001 in NR genes in the maternal Nordic SPTB GWAS data.**

**Table S7. Variants with *p* < 0.0001 in HSP genes in the infant Nordic GWAS of SPTB.**

**Table S8. *HSPA12B* variants with *p* < 0.0001 in the infant Northern Finnish GWAS data.**


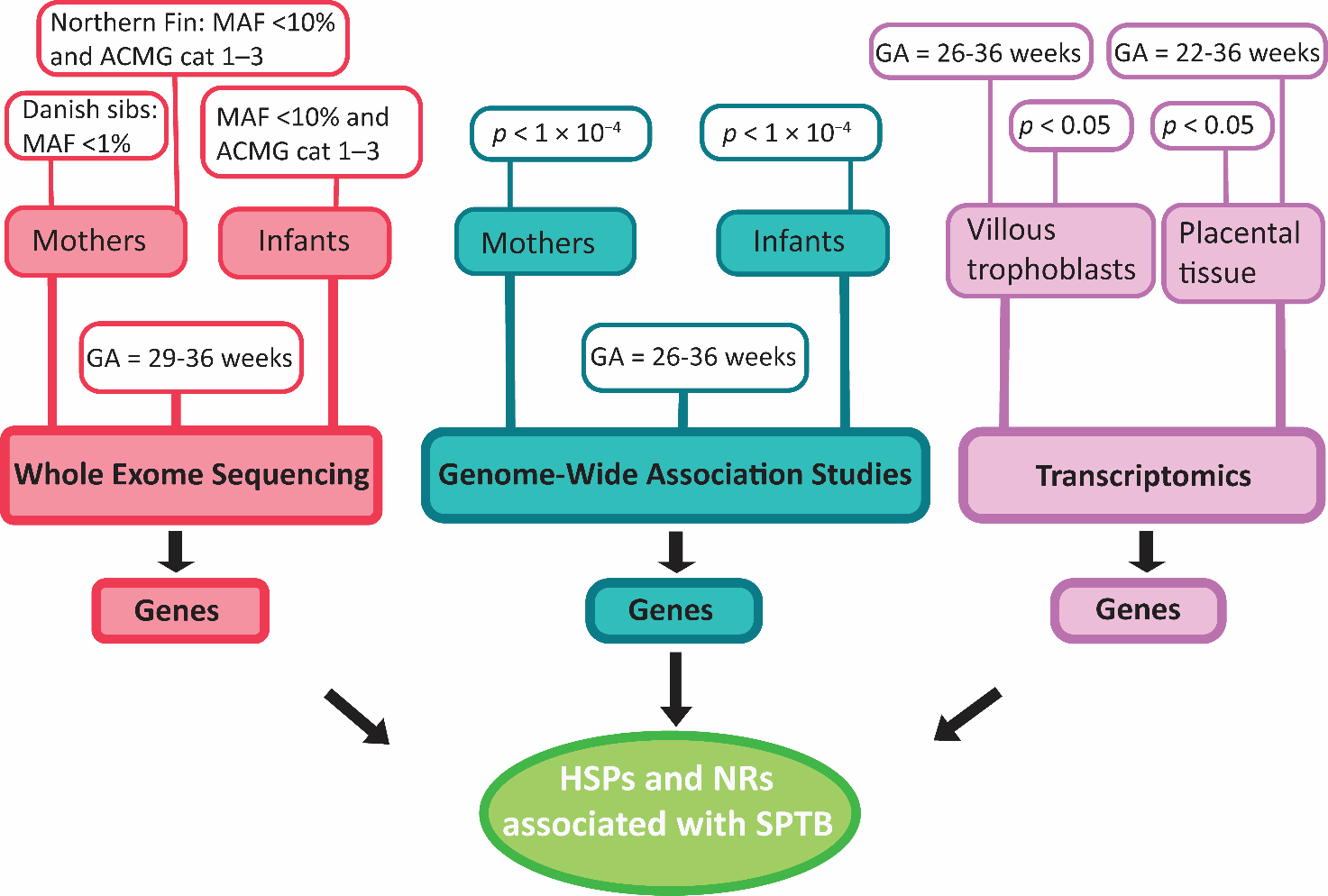


**Figure S1. Overview of the workflow.** Data from whole exome sequencing (WES), genome-wide association study (GWAS) and placental transcriptomic were used in datamining to discover heat shock proteins (HSPs) and nuclear receptors (NRs) associated with spontaneous preterm birth (SPTB). Gestational ages (GA) for each data set are shown. Minor allele frequency (MAF), category 1-3 in accordance with classification of the American College of Medical Genetics (ACMG) and *p*-values were used as cut-offs.

**Table S1. Heat shock protein families and genes.**

| **Group (Family)** | **Gene name** | **Gene ID** | **Chr** | **Gene name (NCBI)** | **Previous gene symbols/synonyms** | **UniProt ID** | **Uniprot name** |
| --- | --- | --- | --- | --- | --- | --- | --- |
| HSP_A (Hsp70) | *HSPA1A* | 3303 | 6 | heat shock protein family A (Hsp70) member 1A | *HSP70-1,HSP72,HSPA1* | P0DMV8 | HS71A |
|  | *HSPA1B* | 3304 | 6 | heat shock protein family A (Hsp70) member 1B | *HSP70-2* | P0DMV9 | HS71B |
|  | *HSPA1L* | 3305 | 6 | heat shock protein family A (Hsp70) member 1 like | *hum70t,Hsp-hom* | P34931 | HS71L |
|  | *HSPA2* | 3306 | 14 | heat shock protein family A (Hsp70) member 2 |  | P54652 | HSP72 |
|  | *HSPA5* | 3309 | 9 | heat shock protein family A (Hsp70) member 5 | *BIP,GRP78,MIF2* | P11021 | GRP78 |
|  | *HSPA6* | 3310 | 1 | heat shock protein family A (Hsp70) member 6 | *HSP70B* | P17066 | HSP76 |
|  | *HSPA7* | 3311 | 1 | heat shock protein family A (Hsp70) member 7 |  | P48741 | HSP77 |
|  | *HSPA8* | 3312 | 11 | heat shock protein family A (Hsp70) member 8 | *HSC70,HSC71,HSP71,HSP73* | P11142 | HSP7C |
|  | *HSPA9* | 3313 | 5 | heat shock protein family A (Hsp70) member 9 | *GRP75,HSPA9B,MOT,MOT2,PBP74* | P38646 | GRP75 |
|  | *HSPA12A* | 259217 | 10 | heat shock protein family A (Hsp70) member 12A | *FLJ13874,KIAA0417* | O43301 | HS12A |
|  | *HSPA12B* | 116835 | 20 | heat shock protein family A (Hsp70) member 12B |  | Q96MM6 | HS12B |
|  | *HSPA13* | 6782 | 21 | heat shock protein family A (Hsp70) member 13 | *Stch* | P48723 | HSP13 |
|  | *HSPA14* | 51182 | 10 | heat shock protein family A (Hsp70) member 14 | *HSP70-4,HSP70L1* | Q0VDF9 | HSP7E |
| HSP_H (110/70) | *HSPH1* | 10808 | 13 | heat shock protein family H (Hsp110) member 1 | *HSP105A,HSP105B,KIAA0201,NY-CO-25* | Q92598 | HS105 |
|  | *HSPA4* | 3308 | 5 | heat shock protein family A (Hsp70) member 4 | *APG-2,HSP110* | P34932 | HSP74 |
|  | *HSPA4L* | 22824 | 4 | heat shock protein family A (Hsp70) member 4 like | *APG-1,Osp94* | O95757 | HS74L |
|  | *HYOU1* | 10525 | 11 | hypoxia up-regulated 1 | *Grp170,ORP150,HSP12A* | Q9Y4L1 | HYOU1 |
| DNAJ_A (Hsp40) | *DNAJA1* | 3301 | 9 | DnaJ heat shock protein family (Hsp40) member A1 | *DJ-2,DjA1,HDJ2,HSDJ,HSJ2,HSPF4* | P31689 | DNJA1 |
|  | *DNAJA2* | 10294 | 16 | DnaJ heat shock protein family (Hsp40) member A2 | *DNJ3,mDj3,Dnaj3,HIRIP4* | O60884 | DNJA2 |
|  | *DNAJA3* | 9093 | 16 | DnaJ heat shock protein family (Hsp40) member A3 | *Tid-1,Tid1l* | Q96EY1 | DNJA3 |
|  | *DNAJA4* | 55466 | 15 | DnaJ heat shock protein family (Hsp40) member A4 | *Dj4,Hsj4,PRO1472* | Q8WW22 | DNJA4 |
| DNAJ_B (Hsp40) | *DNAJB1* | 3337 | 19 | DnaJ heat shock protein family (Hsp40) member B1 | *HSPF1,HSP40,Sis1,RSPH16B* | P25685 | DNJB1 |
|  | *DNAJB2* | 3300 | 2 | DnaJ heat shock protein family (Hsp40) member B2 | *HSJ1,HSPF3,Dnajb10,MDJ8,CMT2T* | P25686 | DNJB2 |
|  | *DNAJB3* | 414061 | 2 | DnaJ heat shock protein family (Hsp40) member B3 | *Hsj3,Msj1,MSJ-1,HCG3* | Q8WWF6 | DNJB3 |
|  | *DNAJB4* | 11080 | 1 | DnaJ heat shock protein family (Hsp40) member B4 | *Hsc40,HLJ1* | Q9UDY4 | DNJB4 |
|  | *DNAJB5* | 25822 | 9 | DnaJ heat shock protein family (Hsp40) member B5 | *Hsc40,HSP40-3* | O75953 | DNJB5 |
|  | *DNAJB6* | 10049 | 7 | DnaJ heat shock protein family (Hsp40) member B6 | *MRJ,mDj4* | O75190 | DNJB6 |
|  | *DNAJB7* | 150353 | 22 | DnaJ heat shock protein family (Hsp40) member B7 | *Dj5,mDj5,HSC3* | Q7Z6W7 | DNJB7 |
|  | *DNAJB8* | 165721 | 3 | DnaJ heat shock protein family (Hsp40) member B8 | *mDj6,CT156* | Q8NHS0 | DNJB8 |
|  | *DNAJB9* | 4189 | 7 | DnaJ heat shock protein family (Hsp40) member B9 | *MDG1,mDj7,ERdj4* | Q9UBS3 | DNJB9 |
|  | *DNAJB11* | 51726 | 3 | DnaJ heat shock protein family (Hsp40) member B11 | *Dj9,ABBP-2,ERdj3,EDJ,HEDJ* | Q9UBS4 | DJB11 |
|  | *DNAJB12* | 54788 | 10 | DnaJ heat shock protein family (Hsp40) member B12 | *DJ10,mDj10* | Q9NXW2 | DJB12 |
|  | *DNAJB13* | 374407 | 11 | DnaJ heat shock protein family (Hsp40) member B13 | *TSARG6,RSPH16A* | P59910 | DJB13 |
|  | *DNAJB14* | 79982 | 4 | DnaJ heat shock protein family (Hsp40) member B14 | *EGNR9427,FLJ14281* | Q8TBM8 | DJB14 |
| DNAJ_C (Hsp40) | *DNAJC1* | 64215 | 10 | DnaJ heat shock protein family (Hsp40) member C1 | *MTJ1,ERdj1,ERj1p,DNAJL1* | Q96KC8 | DNJC1 |
|  | *DNAJC2* | 27000 | 7 | DnaJ heat shock protein family (Hsp40) member C2 | *Zrf1,Zrf2,MIDA1,MPP11* | Q99543 | DNJC2 |
|  | *DNAJC3* | 5611 | 13 | DnaJ heat shock protein family (Hsp40) member C3 | *P58,Prkri,P58IPK,Dnajc3b,HP58,ERdj6* | Q13217 | DNJC3 |
|  | *DNAJC4* | 3338 | 11 | DnaJ heat shock protein family (Hsp40) member C4 | *HSPF2,MCG18* | Q9NNZ3 | DNJC4 |
|  | *DNAJC5* | 80331 | 20 | DnaJ heat shock protein family (Hsp40) member C5 | *Csp,DNAJC5A* | Q9H3Z4 | DNJC5 |
|  | *DNAJC5B* | 85479 | 8 | DnaJ heat shock protein family (Hsp40) member C5 beta | *CSP-beta,MGC6226* | Q9UF47 | DNJ5B |
|  | *DNAJC5G* | 285126 | 2 | DnaJ heat shock protein family (Hsp40) member C5 gamma | *gamma-CSP,FLJ40417* | Q8N7S2 | DNJ5G |
|  | *DNAJC6* | 9829 | 1 | DnaJ heat shock protein family (Hsp40) member C6 | *Auxilin,KIAA0473,PARK19* | O75061 | AUXI |
|  | *DNAJC7* | 7266 | 17 | DnaJ heat shock protein family (Hsp40) member C7 | *TTC2,mDj11,TPR2* | Q99615 | DNJC7 |
|  | *DNAJC8* | 22826 | 1 | DnaJ heat shock protein family (Hsp40) member C8 | *SPF31* | O75937 | DNJC8 |
|  | *DNAJC9* | 23234 | 10 | DnaJ heat shock protein family (Hsp40) member C9 | *RcDNAJ9,JDD1,SB73* | Q8WXX5 | DNJC9 |
|  | *DNAJC10* | 54431 | 2 | DnaJ heat shock protein family (Hsp40) member C10 | *JPDI,ERdj5,PDIA19* | Q8IXB1 | DJC10 |
|  | *DNAJC11* | 55735 | 1 | DnaJ heat shock protein family (Hsp40) member C11 | *FLJ10737,dJ126A5.1* | Q9NVH1 | DJC11 |
|  | *DNAJC12* | 56521 | 10 | DnaJ heat shock protein family (Hsp40) member C12 | *JDP1,mJDP1* | Q9UKB3 | DJC12 |
|  | *DNAJC13* | 23317 | 3 | DnaJ heat shock protein family (Hsp40) member C13 | *RME-8,Gm1124,KIAA0678* | O75165 | DJC13 |
|  | *DNAJC14* | 85406 | 12 | DnaJ heat shock protein family (Hsp40) member C14 | *HDJ3,LIP6,DRIP78,DNAJ,FLJ32792* | Q6Y2X3 | DJC14 |
|  | *DNAJC15* | 29103 | 13 | DnaJ heat shock protein family (Hsp40) member C15 | *Dnajd1,MCJ* | Q9Y5T4 | DJC15 |
|  | *DNAJC16* | 23341 | 1 | DnaJ heat shock protein family (Hsp40) member C16 | *KIAA0962* | Q9Y2G8 | DJC16 |
|  | *DNAJC17* | 55192 | 15 | DnaJ heat shock protein family (Hsp40) member C17 | *C87112,FLJ10634* | Q9NVM6 | DJC17 |
|  | *DNAJC18* | 202052 | 5 | DnaJ heat shock protein family (Hsp40) member C18 | *MGC29463* | Q9H819 | DJC18 |
|  | *DNAJC19* | 131118 | 3 | DnaJ heat shock protein family (Hsp40) member C19 | *TIM14,TIMM14* | Q96DA6 | TIM14 |
|  | *HSCB* | 150274 | 22 | HscB mitochondrial iron-sulfur cluster cochaperone | *JAC1,HSC20,HSCB* | Q8IWL3 | HSC20 |
| DNAJ_C (Hsp40) | *DNAJC21* | 134218 | 5 | DnaJ heat shock protein family (Hsp40) member C21 | *GS3,JJJ1,DNAJA5* | Q5F1R6 | DJC21 |
|  | *DNAJC22* | 79962 | 12 | DnaJ heat shock protein family (Hsp40) member C22 | *FLJ13236,Wurst* | Q8N4W6 | DJC22 |
|  | *SEC63* | 11231 | 6 | SEC63 homolog,protein translocation regulator | *PRO2507,ERdj2,SEC63L* | Q9UGP8 | SEC63 |
|  | *DNAJC24* | 120526 | 11 | DnaJ heat shock protein family (Hsp40) member C24 | *DPH4,ZCSL3,JJJ3* | Q6P3W2 | DJC24 |
|  | *DNAJC25* | 548645 | 9 | DnaJ heat shock protein family (Hsp40) member C25 | *bA16L21.2.1* | Q9H1X3 | DJC25 |
|  | *GAK* | 2580 | 4 | cyclin G associated kinase | *auxilin-2* | O14976 | GAK |
|  | *DNAJC27* | 51277 | 2 | DnaJ heat shock protein family (Hsp40) member C27 | *RBJ,RabJ,RabJS* | Q9NZQ0 | DJC27 |
|  | *DNAJC28* | 54943 | 21 | DnaJ heat shock protein family (Hsp40) member C28 | *Orf28,C21orf78,C21orf55,oculomedin* | Q9NX36 | DJC28 |
|  | *SACS* | 26278 | 13 | sacsin molecular chaperone | *KIAA0730,ARSACS,SPAX6,PPP1R138* | Q9NZJ4 | SACS |
|  | *DNAJC30* | 84277 | 7 | DnaJ heat shock protein family (Hsp40) member C30 | *WBSCR18* | Q96LL9 | DJC30 |
| HSPB (small HSPs) | *HSPB1* | 3315 | 7 | heat shock protein family B (small) member 1 | *CMT2F,HMN2B,HSP27,HSP28,HSP25, HS.76067* | P04792 | HSPB1 |
|  | *HSPB2* | 3316 | 11 | heat shock protein family B (small) member 2 | *MKBP,HSP27,Hs.78846,LOH11CR1K* | Q16082 | HSPB2 |
|  | *HSPB3* | 8988 | 5 | heat shock protein family B (small) member 3 | *HSPL27* | Q12988 | HSPB3 |
|  | *CRYAA* | 1409 | 21 | crystallin alpha A | *CRYA1* | P02489 | CRYAA |
|  | *CRYAB* | 1410 | 11 | crystallin alpha B | *CRYA2* | P02511 | CRYAB |
|  | *HSPB6* | 126393 | 19 | heat shock protein family B (small) member 6 | *HSP20,FLJ32389,PPP1R91* | O14558 | HSPB6 |
|  | *HSPB7* | 27129 | 1 | heat shock protein family B (small) member 7 | *cvHSP,FLJ32733,DKFZp779D0968* | Q9UBY9 | HSPB7 |
|  | *HSPB8* | 26353 | 12 | heat shock protein family B (small) member 8 | *H11,HMN2,CMT2L,DHMN2,E2IG1,HMN2A, HSP22,Hsp88* | Q9UJY1 | HSPB8 |
|  | *HSPB9* | 94086 | 17 | heat shock protein family B (small) member 9 | *FLJ27437,CT51* | Q9BQS6 | HSPB9 |
|  | *ODF1* | 4956 | 8 | outer dense fiber of sperm tails 1 | *RT7,ODF2,ODFP,SODF,ODF,ODF27,ODFPG,ODFPGA,ODFPGB,CT133* | Q14990 | ODFP1 |
|  | *HSPB11* | 51668 | 1 | heat shock protein family B (small) member 11 | *HSP16.2,C1orf41,PP25* | Q9Y547 | IFT25 |
| HSPC (Hsp90) | *HSP90AA1* | 3320 | 14 | heat shock protein 90 alpha family class A member 1 | *HSPN,HSP86,HSPC1,HSPCA,LAP2,HSP89,HSP90,HSP90A,HSP90N,HSPCAL1,HSPCAL4* | P07900 | HS90A |
|  | *HSP90AA2P* | 3324 | 11 | heat shock protein 90 alpha family class A member 2, pseudogene | *HSPCA,HSPCAL3,HSP90ALPHA* | Q14568 | HS902 |
|  | *HSP90AB1* | 3326 | 6 | heat shock protein 90 alpha family class B member 1 | *HSPC2,HSPCB,D6S182,HSP90B,FLJ26984* | P08238 | HS90B |
|  | *HSP90B1* | 7184 | 12 | heat shock protein 90 beta family member 1 | *ECGP,GP96,TRA1,GRP94* | P14625 | ENPL |
|  | *TRAP1* | 10131 | 16 | TNF receptor associated protein 1 | *HSP75,HSP90L* | Q12931 | TRAP1 |
| HSPD,HSPE,CCT (chaperonins) | *HSPD1* | 3329 | 2 | heat shock protein family D (Hsp60) member 1 | *HSP60,GroEL,SPG13* | P10809 | CH60 |
|  | *HSPE1* | 3336 | 2 | heat shock protein family E (Hsp10) member 1 | *HSP10,GroES,CPN10,EPF* | P61604 | CH10 |
|  | *TCP1* | 6950 | 6 | t-complex 1 | *CCTA,TCP-1A,D6S230E* | P17987 | TCPA |
|  | *CCT2* | 10576 | 12 | chaperonin containing TCP1 subunit 2 | *CCTB,TCP-1B* | P78371 | TCPB |
|  | *CCT3* | 7203 | 1 | chaperonin containing TCP1 subunit 3 | *CCTG,TCP-1G,TRiC-P5,TRIC5* | P49368 | TCPG |
|  | *CCT4* | 10575 | 2 | chaperonin containing TCP1 subunit 4 | *CCTD,TCP-1D,SRB* | P50991 | TCPD |
|  | *CCT5* | 22948 | 5 | chaperonin containing TCP1 subunit 5 | *CCTE,TCP-1E,KIAA0098* | P48643 | TCPE |
|  | *CCT6A* | 908 | 7 | chaperonin containing TCP1 subunit 6A | *CCT6,CCTZ,CCT-Z1,HTR3,TCP20,TTCP20* | P40227 | TCPZ |
|  | *CCT6B* | 10693 | 17 | chaperonin containing TCP1 subunit 6B | *CCTZ2,TSA303* | Q92526 | TCPW |
|  | *CCT7* | 10574 | 2 | chaperonin containing TCP1 subunit 7 | *CCTH,TCP-1-eta,NIP7-1* | Q99832 | TCPH |
|  | *CCT8* | 10694 | 21 | chaperonin containing TCP1 subunit 8 | *CCTQ,PRED71* | Q94K05 | TCPQ |
|  | *CLPB* | 81570 | 11 | ClpB homolog, mitochondrial AAA ATPase chaperonin | *HSP78,SKD3,ANKCLB* | Q9H078 | CLPB |
| other chaperonin-like | *MKKS* | 8195 | 20 | McKusick-Kaufman syndrome | *MKS,BBS6* | Q9NPJ1 | MKKS |
|  | *BBS10* | 79738 | 12 | Bardet-Biedl syndrome 10 | *C12orf58,FLJ23560* | Q8TAM1 | BBS10 |
|  | *BBS12* | 166379 | 4 | Bardet-Biedl syndrome 12 | *C4orf24,FLJ35630* | Q6ZW61 | BBS12 |

**Table S2. Nuclear hormone receptor (NR) genes.**

| **Gene** | **GeneID** | **Chr** | **Name** | **Previous gene symbols/Synonyms** | **UniProt ID** | **Uniprot name** |
| --- | --- | --- | --- | --- | --- | --- |
| *AR* | 367 | X | androgen receptor | *DHTR,SBMA,AIS,SMAX1,HUMARA,NR3C4* | P10275 | ANDR |
| *ESR1* | 2099 | 6 | estrogen receptor 1 | *ESR,ER-alpha,NR3A1,Era* | P03372 | ESR1 |
| *ESR2* | 2100 | 14 | estrogen receptor 2 | *ER-beta,NR3A2,Erb* | Q92731 | ESR2 |
| *ESRRA* | 2101 | 11 | estrogen related receptor alpha | *ESRL1,NR3B1,ERRalpha,ERRa,ERR1* | P11474 | ERR1 |
| *ESRRB* | 2103 | 14 | estrogen related receptor beta | *ESRL2,DFNB35,ERR2,NR3B2,ERRb,ERRbeta* | O95718 | ERR2 |
| *ESRRG* | 2104 | 1 | estrogen related receptor gamma | *NR3B3,ERR-gamma,ERRg* | P62508 | ERR3 |
| *HNF4A* | 3172 | 20 | hepatocyte nuclear factor 4 alpha | *TCF14,MODY,MODY1,HNF4,NR2A1* | P41235 | HNF4A |
| *HNF4G* | 3174 | 8 | hepatocyte nuclear factor 4 gamma | *NR2A2* | Q14541 | HNF4G |
| *NR0B1* | 190 | X | nuclear receptor subfamily 0 group B member 1 | *AHC,DSS,DAX1,AHCH* | P51843 | NR0B1 |
| *NR0B2* | 8431 | 1 | nuclear receptor subfamily 0 group B member 2 | *SHP* | Q15466 | NR0B2 |
| *NR1D1* | 9572 | 17 | nuclear receptor subfamily 1 group D member 1 | *THRAL,ear-1,hRev,Rev-ErbAalpha,THRA1,REVERBalpha* | P20393 | NR1D1 |
| *NR1D2* | 9975 | 3 | nuclear receptor subfamily 1 group D member 2 | *BD73,RVR,EAR-1r,HZF2,Hs.37288,REVERBbeta* | Q14995 | NR1D2 |
| *NR1H2* | 7376 | 19 | nuclear receptor subfamily 1 group H member 2 | *UNR,NER,NER-I,RIP15,LXR-b,LXRb* | P55055 | NR1H2 |
| *NR1H3* | 10062 | 11 | nuclear receptor subfamily 1 group H member 3 | *LXR-a,RLD-1,LXRa* | Q13133 | NR1H3 |
| *NR1H4* | 9971 | 12 | nuclear receptor subfamily 1 group H member 4 | *FXR,RIP14,HRR1,HRR-1* | Q96RI1 | NR1H4 |
| *NR1H5P* | 643609 | 1 | nuclear receptor subfamily 1 group H member 5, pseudogene | *Fxrb,NR1H5* | N/A | N/A |
| *NR1I2* | 8856 | 3 | nuclear receptor subfamily 1 group I member 2 | *ONR1,PXR,BXR,SXR,PAR2* | O75469 | NR1I2 |
| *NR1I3* | 9970 | 1 | nuclear receptor subfamily 1 group I member 3 | *MB67,CAR1,CAR* | Q14994 | NR1I3 |
| *NR2C1* | 7181 | 12 | nuclear receptor subfamily 2 group C member 1 | *TR2,TR2-11* | P13056 | NR2C1 |
| *NR2C2* | 7182 | 3 | nuclear receptor subfamily 2 group C member 2 | *TR4,TAK1,TR2R1,hTAK1* | P49116 | NR2C2 |
| *NR2E1* | 7101 | 6 | nuclear receptor subfamily 2 group E member 1 | *TLX,TLL,XTLL* | Q9Y466 | NR2E1 |
| *NR2E3* | 10002 | 15 | nuclear receptor subfamily 2 group E member 3 | *PNR,rd7,RP37* | Q9Y5X4 | NR2E3 |
| *NR2F1* | 7025 | 5 | nuclear receptor subfamily 2 group F member 1 | *ERBAL3,TFCOUP1,EAR-3,COUP-TFI, TCFCOUP1, SVP44, COUPTF1* | P10589 | COT1 |
| *NR2F2* | 7026 | 15 | nuclear receptor subfamily 2 group F member 2 | *ARP1,TFCOUP2,COUP-TFII,COUPTFB,SVP40,NF-E3, COUPTF2* | P24468 | COT2 |
| *NR2F6* | 2063 | 19 | nuclear receptor subfamily 2 group F member 6 | *ERBAL2,EAR-2,EAR2* | P10588 | NR2F6 |
| *NR3C1* | 2908 | 5 | nuclear receptor subfamily 3 group C member 1 | *GRL,GR* | P04150 | GCR |
| *NR3C2* | 4306 | 4 | nuclear receptor subfamily 3 group C member 2 | *MLR,MR* | P08235 | MCR |
| *NR4A1* | 3164 | 12 | nuclear receptor subfamily 4 group A member 1 | *HMR,GFRP1,TR3,N10,NAK-1,NGFIB,NUR77* | P22736 | NR4A1 |
| *NR4A2* | 4929 | 2 | nuclear receptor subfamily 4 group A member 2 | *NURR1,TINUR,NOT,RNR1,HZF-3* | P43354 | NR4A2 |
| *NR4A3* | 8013 | 9 | nuclear receptor subfamily 4 group A member 3 | *CSMF,CHN,NOR1,MINOR* | Q92570 | NR4A3 |
| *NR5A1* | 2516 | 9 | nuclear receptor subfamily 5 group A member 1 | *FTZF1,FTZ1,SF-1,ELP,AD4BP,hSF-1,SF1* | Q13285 | STF1 |
| *NR5A2* | 2494 | 1 | nuclear receptor subfamily 5 group A member 2 | *FTF,FTZ-F1beta,hB1F,LRH-1,FTZ-F1,hB1F-2,B1F2,LRH1* | O00482 | NR5A2 |
| *NR6A1* | 2649 | 9 | nuclear receptor subfamily 6 group A member 1 | *GCNF,GCNF1,RTR,CT150* | Q15406 | NR6A1 |
| *PGR* | 5241 | 11 | progesterone receptor | *PR,NR3C3* | P06401 | PRGR |
| *PPARA* | 5465 | 22 | peroxisome proliferator activated receptor alpha | *PPAR,hPPAR,NR1C1* | Q07869 | PPARA |
| *PPARD* | 5467 | 6 | peroxisome proliferator activated receptor delta | *NUCII,FAAR,NUC1,NR1C2* | Q03181 | PPARD |
| *PPARG* | 5468 | 3 | peroxisome proliferator activated receptor gamma | *NR1C3,PPARG2,PPARgamma,PPARG1* | P37231 | PPARG |
| *RARA* | 5914 | 17 | retinoic acid receptor alpha | *NR1B1,RAR* | P10276 | RARA |
| *RARB* | 5915 | 3 | retinoic acid receptor beta | *HAP,RRB2,NR1B2* | P10826 | RARB |
| *RARG* | 5916 | 12 | retinoic acid receptor gamma | *RARC,NR1B3* | P13631 | RARG |
| *RORA* | 6095 | 15 | RAR related orphan receptor A | *NR1F1,ROR1,ROR3,ROR2,RZRA* | P35398 | RORA |
| *RORB* | 6096 | 9 | RAR related orphan receptor B | *RZRB,ROR-BETA,NR1F2* | Q92753 | RORB |
| *RORC* | 6097 | 1 | RAR related orphan receptor C | *RORG,RZRG,NR1F3,TOR* | P51449 | RORG |
| *RXRA* | 6256 | 9 | retinoid X receptor alpha | *NR2B1* | P19793 | RXRA |
| *RXRB* | 6257 | 6 | retinoid X receptor beta | *RCoR-1,H-2RIIBP,NR2B2* | P28702 | RXRB |
| *RXRG* | 6258 | 1 | retinoid X receptor gamma | *NR2B3* | P48443 | RXRG |
| *THRA* | 7067 | 17 | thyroid hormone receptor alpha | *THRA1,THRA2,ERBA1,NR1A1,EAR-7.1/EAR-7.2, AR7, THRA3,ERBA* | P10827 | THA |
| *THRB* | 7068 | 3 | thyroid hormone receptor beta | *ERBA2,PRTH,ERBA-BETA,THR1,GRTH, NR1A2, THRB2, THRB1* | P10828 | THB |
| *VDR* | 7421 | 12 | vitamin D receptor | *PPP1R163,NR1I1* | P11473 | VDR |

**Table S3. Variants with *p* < 0.0001 in HSP gene regions +/- 100 kb in maternal 23andMe SPTB GWAS data.**

| **Gene^1^** | **Rs** | **Chr** | **Pos** | **Alleles** | ***p* Value** | **Freq** |
| --- | --- | --- | --- | --- | --- | --- |
| ***DNAJB8*** | rs1735545 | 3 | 128081260 | C/G | 4.32E-09 | 0.771 |
|  | rs1702122 | 3 | 128083332 | A/G | 5.53E-09 | 0.255 |
|  | rs1702119 | 3 | 128094493 | C/T | 6.21E-09 | 0.746 |
|  | rs1735538 | 3 | 128092102 | A/G | 6.34E-09 | 0.255 |
|  | rs2977565 | 3 | 128095767 | A/G | 6.63E-09 | 0.772 |
|  | rs3021461 | 3 | 128095652 | C/T | 6.69E-09 | 0.772 |
|  | rs7632169 | 3 | 128114587 | C/T | 5.05E-08 | 0.744 |
|  | rs4241495 | 3 | 128111174 | C/T | 6.32E-08 | 0.744 |
|  | rs1702136 | 3 | 128118711 | A/G | 6.93E-08 | 0.769 |
|  | rs760383 | 3 | 128119565 | A/G | 7.56E-08 | 0.231 |
|  | rs2659685 | 3 | 128122396 | A/G | 7.68E-08 | 0.756 |
|  | rs1625296 | 3 | 128107982 | A/G | 8.32E-08 | 0.744 |
|  | rs729847 | 3 | 128110770 | A/G | 9.21E-08 | 0.770 |
|  | rs2977561 | 3 | 128106536 | C/T | 1.13E-07 | 0.745 |
|  | rs3887841 | 3 | 128109941 | C/T | 1.19E-07 | 0.771 |
|  | rs2977562 | 3 | 128106267 | A/G | 1.21E-07 | 0.255 |
|  | rs7373685 | 3 | 128107020 | A/C | 1.22E-07 | 0.255 |
|  | rs4277707 | 3 | 128084164 | A/T | 7.19E-07 | 0.139 |
|  | rs1702118 | 3 | 128095278 | G/T | 1.01E-06 | 0.861 |
|  | rs1735537 | 3 | 128122820 | C/T | 1.77E-06 | 0.758 |
|  | rs1735527 | 3 | 128115381 | A/G | 2.99E-06 | 0.127 |
|  | rs1702153 | 3 | 128117573 | A/G | 3.75E-06 | 0.875 |
|  | rs2999033 | 3 | 128105972 | A/G | 4.06E-06 | 0.873 |
|  | rs1620440 | 3 | 128112307 | C/T | 4.21E-06 | 0.874 |
|  | rs1702134 | 3 | 128111201 | G/T | 4.32E-06 | 0.126 |
|  | rs1702131 | 3 | 128110390 | C/T | 4.35E-06 | 0.874 |
|  | rs741925 | 3 | 128109916 | C/T | 5.05E-06 | 0.124 |
|  | rs6780368 | 3 | 128122039 | C/T | 7.77E-06 | 0.125 |
|  | rs1108313 | 3 | 128085090 | A/G | 1.25E-05 | 0.538 |
|  | rs2977564 | 3 | 128123786 | A/G | 2.51E-05 | 0.602 |
|  | rs2981017 | 3 | 128113829 | C/T | 4.55E-05 | 0.438 |
|  | rs2999031 | 3 | 128121502 | A/T | 7.48E-05 | 0.561 |
| ***DNAJB14*** | rs75110318 | 4 | 100738592 | C/T | 2.95E-06 | 0.916 |
|  | rs62305027 | 4 | 100731832 | C/G | 3.25E-06 | 0.084 |
|  | rs62305030 | 4 | 100734069 | C/G | 3.29E-06 | 0.916 |
|  | rs62305031 | 4 | 100745110 | A/G | 3.29E-06 | 0.084 |
|  | rs80062446 | 4 | 100746346 | A/C | 3.44E-06 | 0.916 |
|  | rs56212993 | 4 | 100749036 | A/G | 3.66E-06 | 0.916 |
|  | rs138883508 | 4 | 100729996 | D/I | 6.34E-06 | 0.916 |
|  | rs62305026 | 4 | 100729914 | C/T | 6.96E-06 | 0.084 |
| ***DNAJC6*** | rs11208607 | 1 | 65668481 | A/G | 3.68E-06 | 0.362 |
|  | rs4916030 | 1 | 65668191 | C/T | 4.34E-06 | 0.342 |
|  | rs10889526 | 1 | 65667802 | C/T | 4.72E-06 | 0.383 |
|  | rs35119851 | 1 | 65668616 | D/I | 4.90E-06 | 0.657 |
|  | rs34696356 | 1 | 65667903 | D/I | 5.11E-06 | 0.342 |
|  | rs11208608 | 1 | 65668543 | C/T | 6.59E-06 | 0.635 |
|  | rs4915684 | 1 | 65670441 | A/G | 7.23E-06 | 0.343 |
|  | rs10889525 | 1 | 65664249 | C/T | 8.76E-06 | 0.661 |
|  | rs2051092 | 1 | 65663384 | A/T | 8.80E-06 | 0.338 |
|  | rs7526688 | 1 | 65674836 | A/G | 9.29E-06 | 0.656 |
|  | rs7553495 | 1 | 65663804 | C/G | 9.52E-06 | 0.660 |
|  | rs10465810 | 1 | 65673646 | A/T | 9.77E-06 | 0.394 |
|  | rs12042230 | 1 | 65672432 | A/T | 1.05E-05 | 0.636 |
|  | rs10889527 | 1 | 65675857 | A/G | 1.09E-05 | 0.344 |
|  | rs12743435 | 1 | 65678848 | A/G | 1.32E-05 | 0.344 |
|  | rs199853266 | 1 | 65665721 | D/I | 1.42E-05 | 0.363 |
|  | rs10633741 | 1 | 65665722 | D/I | 1.42E-05 | 0.363 |
|  | rs12568459 | 1 | 65678454 | A/G | 1.51E-05 | 0.344 |
|  | rs147357021 | 1 | 65676920 | D/I | 2.34E-05 | 0.665 |
|  | rs11208610 | 1 | 65679229 | A/G | 2.51E-05 | 0.665 |
|  | rs12561779 | 1 | 65676839 | A/G | 2.62E-05 | 0.335 |
|  | rs11208609 | 1 | 65678419 | C/G | 2.75E-05 | 0.335 |
|  | rs10789175 | 1 | 65662981 | A/G | 2.79E-05 | 0.334 |
|  | rs10789176 | 1 | 65677933 | C/T | 2.88E-05 | 0.335 |
|  | rs11579215 | 1 | 65680623 | C/G | 3.53E-05 | 0.335 |
|  | rs7516366 | 1 | 65662117 | C/T | 5.24E-05 | 0.692 |
|  | rs6700762 | 1 | 65654456 | C/T | 8.64E-05 | 0.674 |
|  | rs4916028 | 1 | 65652891 | A/G | 8.92E-05 | 0.326 |
|  | rs34985293 | 1 | 65654059 | D/I | 9.19E-05 | 0.326 |
|  | rs4511159 | 1 | 65661665 | A/G | 9.98E-05 | 0.668 |
| ***DNAJA3*** | rs11648292 | 16 | 4462897 | A/C | 7.46E-05 | 0.307 |
|  | rs4640182 | 16 | 4471291 | A/C | 8.04E-05 | 0.754 |
|  | rs6500596 | 16 | 4470027 | G/T | 8.98E-05 | 0.754 |
|  | rs57674835 | 16 | 4464015 | C/T | 9.10E-05 | 0.246 |
|  | rs934888 | 16 | 4462832 | C/T | 9.25E-05 | 0.754 |
|  | rs6500598 | 16 | 4470964 | G/T | 9.93E-05 | 0.753 |
| ***SEC63*** | **rs200623075** | 6 | 108229548 | D/I | 9.98E-05 | 0.957 |

SNPs within the gene body are in boldface.

^1^*HSP* gene regions in GRCh37.p13 coordinates: ***DNAJB8*** Chr3:128,181,275–128,186,091; ***DNAJB14*** Chr4:100,817,405–100,867,883; ***DNAJC6*** Chr1:65,720,133–65,881,552; ***DNAJA3*** Chr16:4,475,806–4,506,776; ***SEC63*** Chr6:108,188,960–108,279,482.

**Table S4. Variants with *p* < 0.0001 in HSP genes +/- 100 kb in the maternal Nordic SPTB GWAS data.**

| **Dataset^1^** | **Gene^2^** | **Rs** | **Chr** | **Pos** | **a1** | **a2** | **Freq.** | **Eff.** | **se** | ***p* Value** |
| --- | --- | --- | --- | --- | --- | --- | --- | --- | --- | --- |
| **FIN**  ***n* = 888** | ***MKKS*** | **rs6108571** | 20 | **10410320** | G | A | 0.5998 | 1.4997 | 0.1038 | 9.43E-05 |
|  |  | **rs17452140** | 20 | **10410253** | T | C | 0.5998 | 1.4997 | 0.1038 | 9.43E-05 |
|  |  | **rs6133929** | 20 | **10410080** | T | C | 0.5996 | 1.4998 | 0.1038 | 9.47E-05 |
|  |  | **rs6077783** | 20 | **10410431** | G | A | 0.5998 | 1.4995 | 0.1038 | 9.49E-05 |
|  |  | **rs6077784** | 20 | **10410534** | A | G | 0.5998 | 1.4994 | 0.1038 | 9.55E-05 |
|  |  | **rs33986973** | 20 | **10410686** | I | D | 0.5998 | 1.4995 | 0.1038 | 9.57E-05 |
|  |  | **rs6039924** | 20 | **10410968** | G | T | 0.5998 | 1.4994 | 0.1039 | 9.63E-05 |
|  |  | **rs33959941** | 20 | **10411071** | D | I | 0.5997 | 1.4994 | 0.1039 | 9.66E-05 |
| **MoBa**  ***n* = 1834** | ***DNAJA1*** | rs1537254 | 9 | 33004339 | T | C | 0.7415 | 1.3575 | 0.0775 | 8.11E-05 |
|  |  | rs13297493 | 9 | 33049601 | A | C | 0.7426 | 1.3565 | 0.0775 | 8.28E-05 |
|  |  | rs7041702 | 9 | 33053430 | A | G | 0.7431 | 1.3555 | 0.0774 | 8.55E-05 |
|  |  | **rs4879659** | 9 | **33029468** | G | C | 0.7432 | 1.3548 | 0.0774 | 8.71E-05 |
|  |  | **rs4879662** | 9 | **33035566** | C | T | 0.7432 | 1.3548 | 0.0774 | 8.71E-05 |
|  |  | **rs10971347** | 9 | **33031138** | G | A | 0.7432 | 1.3548 | 0.0774 | 8.71E-05 |
|  |  | **rs4879660** | 9 | **33032833** | A | G | 0.7432 | 1.3548 | 0.0774 | 8.71E-05 |
|  |  | **rs10448231** | 9 | **33034051** | A | G | 0.7432 | 1.3548 | 0.0774 | 8.71E-05 |
|  |  | rs72527606 | 9 | 33001178 | I | D | 0.7432 | 1.3547 | 0.0774 | 8.72E-05 |
|  |  | rs10971307 | 9 | 33009724 | C | T | 0.7432 | 1.3547 | 0.0774 | 8.74E-05 |
|  |  | rs10971308 | 9 | 33009725 | C | T | 0.7432 | 1.3547 | 0.0774 | 8.74E-05 |
|  |  | rs3758276 | 9 | 33024917 | G | A | 0.7432 | 1.3546 | 0.0774 | 8.78E-05 |
|  |  | rs10971397 | 9 | 33087805 | G | C | 0.7093 | 1.3384 | 0.0745 | 9.09E-05 |
|  |  | rs10971398 | 9 | 33087938 | C | T | 0.7093 | 1.3384 | 0.0745 | 9.11E-05 |
|  |  | rs11560521 | 9 | 33088439 | A | G | 0.7093 | 1.3381 | 0.0745 | 9.18E-05 |
|  |  | rs13285805 | 9 | 33091249 | T | C | 0.7093 | 1.3369 | 0.0744 | 9.52E-05 |
|  | ***DNAJC17*** | rs627458 | 15 | 41197736 | T | C | 0.9556 | 1.9884 | 0.1735 | 7.47E-05 |
|  |  | rs604751 | 15 | 41198894 | A | G | 0.9556 | 1.9882 | 0.1735 | 7.48E-05 |
|  |  | rs3100812 | 15 | 41199503 | C | G | 0.9556 | 1.9881 | 0.1735 | 7.49E-05 |
| **DNBC**  ***n* = 1910** | ***DNAJB14*** | **rs17613664** | **4** | **100851879** | C | T | 0.9356 | 1.857 | 0.1562 | 7.35E-05 |
|  | ***DNAJC2*** | rs189506709 | 7 | 102895539 | T | C | 0.9835 | 4.7249 | 0.3882 | 6.33E-05 |
| **Metadata**  ***n* = 4632** | ***DNAJB8^3^*** | rs2659685 | 3 | 128122396 | G | A | 0.7695 | 1.242 | 0.0511 | 2.23E-05 |
|  |  | rs1735537 | 3 | 128122820 | T | C | 0.7685 | 1.240 | 0.0508 | 2.36E-05 |
|  |  | rs113924139 | 3 | 128122539 | - | CCCAC | 0.7813 | 1.240 | 0.0520 | 3.57E-05 |
|  |  | rs760383 | 3 | 128119565 | A | G | 0.7793 | 1.233 | 0.0517 | 5.22E-05 |
|  |  | rs1702136 | 3 | 128118711 | G | A | 0.7797 | 1.231 | 0.0518 | 5.86E-05 |
|  |  | rs3887841 | 3 | 128109941 | T | C | 0.7795 | 1.229 | 0.0517 | 6.82E-05 |
|  |  | rs729847 | 3 | 128110770 | G | A | 0.7796 | 1.229 | 0.0517 | 6.88E-05 |
|  | ***DNAJC1*** | rs72802918 | 10 | 21982459 | C | G | 0.9086 | 0.7459 | 0.0752 | 9.62E-05 |
|  | ***DNAJC11*** | rs10864636 | 1 | 6799214 | G | A | 0.7664 | 0.7699 | 0.0611 | 1.87E-05 |

SNPs located within the gene body are in boldface.

^1^Subdatasets of the Nordic data; FIN = Finnish, MoBa = Norwegian, DNBC = Danish. ^2^*HSP* gene regions in GRCh37.p13 coordinates: ***MKKS*** Chr20:10,385,428–10,414,887; ***DNAJA1*** Chr9:33,025,209–33,039,905; ***DNAJC17*** Chr15:41,060,067–41,099,676; ***DNAJB14*** Chr4:100,817,405–100,867,883; ***DNAJC2*** Chr7:102,952,921–102,985,320; ***DNAJB8*** Chr3:128,181,275–128,186,091; ***DNAJC1*** Chr10:22,045,477–22,292,679; ***DNAJC11*** Chr1:6,694,228–6,761,966. ^3^All SNPs, except rs113924139, in *DNAJB8* had *p*<0.0001 in the 23andMe data.

**Table S5. Variants with *p <* 0.0001 in NR genes in the maternal 23andMe SPTB GWAS data.**

| **Gene^1^** | **Rs** | **Chr** | **Pos** | **Alleles** | **Freq** | ***p* Value** |
| --- | --- | --- | --- | --- | --- | --- |
| ***NR2F2*** | rs140287246 | 15 | 96,917,459 | C/T | 0.021 | 1.41E-06 |
| ***THRA*** | rs55739615 | 17 | 38,119,638 | C/T | 0.562 | 4.62E-06 |
|  | rs3902025 | 17 | 38,119,254 | G/T | 0.563 | 4.99E-06 |
|  | rs2001476 | 17 | 38,120,604 | C/T | 0.372 | 1.33E-05 |
|  | rs56396280 | 17 | 38,119,708 | A/C | 0.628 | 1.42E-05 |
|  | rs3902024 | 17 | 38,119,548 | A/G | 0.628 | 1.46E-05 |
|  | rs11869855 | 17 | 38,117,653 | A/G | 0.628 | 1.48E-05 |
|  | rs4458030 | 17 | 38,121,706 | A/G | 0.372 | 1.68E-05 |
|  | rs12943476 | 17 | 38,121,514 | A/G | 0.605 | 1.78E-05 |
|  | rs34670005 | 17 | 38,216,933 | C/T | 0.637 | 3.23E-05 |
|  | **rs7502233** | 17 | **38,218,804** | A/G | 0.346 | 3.35E-05 |
|  | rs199873165 | 17 | 38,186,918 | A/- | 0.629 | 3.54E-05 |
|  | rs5820320 | 17 | 38,186,917 | -/A | 0.371 | 4.12E-05 |
|  | rs72045810 | 17 | 38,186,916 | -/T/TA | 0.371 | 4.13E-05 |
|  | **rs7502539** | 17 | **38,219,005** | A/G | 0.652 | 4.71E-05 |
|  | rs4072639 | 17 | 38,189,049 | A/G | 0.62 | 6.96E-05 |
|  | rs3935281 | 17 | 38,188,419 | C/T | 0.38 | 7.03E-05 |
|  | rs36071767 | 17 | 38,186,825 | C/T | 0.62 | 7.88E-05 |
|  | rs8078692 | 17 | 38,215,117 | A/G | 0.508 | 7.99E-05 |
|  | rs7502971 | 17 | 38,186,501 | A/C | 0.62 | 8.28E-05 |
| ***PPARG*** | rs7637403 | 3 | 12,242,488 | A/G | 0.879 | 2.64E-05 |
|  | rs13079837 | 3 | 12,250,846 | A/T | 0.877 | 2.90E-05 |
|  | rs13099882 | 3 | 12,250,888 | A/G | 0.123 | 3.16E-05 |
|  | **rs112215093** | 3 | **12,366,204** | A/G | 0.964 | 9.16E-05 |
| ***RORA*** | rs72741635 | 15 | 61,598,417 | A/G | 0.945 | 5.93E-05 |

SNPs within the gene body are in boldface.

^1^*NR* gene regions in GRCh37.p13 coordinates: ***NR2F2*** Chr15:96,869,157–96,883,492; ***THRA*** Chr17:38,218,446–38,250,120; ***PPARG*** Chr3:12,329,349–12,475,855; ***RORA*** Chr15:60,780,483–61,521,502

**Table S6. Variants with *p <* 0.0001 in NR genes in the maternal Nordic SPTB GWAS data.**

| **Dataset^1^** | **Gene^2^** | **Rs** | **Pos** | **a1** | **a2** | ***n*** | **Freq.** | **Eff.** | **se.** | **p Value** |
| --- | --- | --- | --- | --- | --- | --- | --- | --- | --- | --- |
| **MoBa**  ***n* = 1834** | ***RORA*** | **rs8042259** | **61,385,824** | C | T | 1834 | 0.5881 | 1.3455 | 0.0686 | 1.52E-05 |
|  |  | **rs1437535** | **61,385,177** | C | T | 1834 | 0.582 | 1.3372 | 0.0683 | 2.12E-05 |
|  |  | **rs1437537** | **61,385,274** | C | T | 1834 | 0.582 | 1.3372 | 0.0684 | 2.13E-05 |
|  |  | **rs893288** | **61,394,450** | T | C | 1834 | 0.5834 | 1.326 | 0.068 | 3.33E-05 |
|  |  | **rs893287** | **61,394,232** | G | A | 1834 | 0.5917 | 1.3188 | 0.0685 | 5.39E-05 |
|  |  | **rs11071586** | **61,378,086** | T | C | 1834 | 0.5789 | 1.3136 | 0.0678 | 5.77E-05 |
| **FIN**  ***n* = 888** | ***ESR1*** | rs542215671 | 151,946,173 | I | D | 888 | 0.8068 | 0.5898 | 0.1212 | 1.33E-05 |
|  |  | rs6929137 | 151,936,677 | G | A | 888 | 0.8162 | 0.5884 | 0.1232 | 1.68E-05 |
|  |  | rs11155802 | 151,945,470 | C | T | 888 | 0.8166 | 0.5889 | 0.1232 | 1.71E-05 |
|  |  | rs11155803 | 151,945,669 | T | C | 888 | 0.8166 | 0.5889 | 0.1231 | 1.71E-05 |
|  |  | rs11155805 | 151,947,326 | A | G | 888 | 0.8165 | 0.5893 | 0.1231 | 1.73E-05 |
|  |  | rs7763637 | 151,949,312 | G | A | 888 | 0.8165 | 0.5897 | 0.123 | 1.77E-05 |
|  |  | rs6557160 | 151,949,582 | A | C | 888 | 0.8165 | 0.5897 | 0.123 | 1.77E-05 |
|  |  | rs7740686 | 151,948,173 | A | T | 888 | 0.8164 | 0.5898 | 0.123 | 1.78E-05 |
|  |  | rs3734806 | 151,941,461 | G | A | 888 | 0.8164 | 0.5898 | 0.1232 | 1.81E-05 |
|  |  | rs3757322 | 151,942,194 | T | G | 888 | 0.8164 | 0.5899 | 0.1232 | 1.82E-05 |
|  |  | rs6913578 | 151,949,806 | A | C | 888 | 0.8187 | 0.5895 | 0.1237 | 1.94E-05 |
|  |  | rs75859313 | 151,935,539 | G | C | 888 | 0.7987 | 0.6082 | 0.1193 | 3.06E-05 |
|  |  | rs6900157 | 151,954,127 | T | C | 888 | 0.8023 | 0.614 | 0.1192 | 4.28E-05 |
|  |  | rs11155804 | 151,946,152 | T | A | 888 | 0.7996 | 0.6155 | 0.1187 | 4.33E-05 |
|  |  | rs2046210 | 151,948,366 | G | A | 888 | 0.7995 | 0.6162 | 0.1186 | 4.47E-05 |
|  |  | rs60705924 | 151,955,985 | A | G | 888 | 0.8035 | 0.6136 | 0.1197 | 4.49E-05 |
|  |  | rs6930633 | 151,958,091 | A | G | 888 | 0.8035 | 0.6138 | 0.1197 | 4.58E-05 |
|  |  | rs58164038 | 151,956,201 | A | G | 888 | 0.8045 | 0.6131 | 0.1202 | 4.72E-05 |
|  |  | rs6557161 | 151,950,235 | A | G | 888 | 0.8018 | 0.616 | 0.1192 | 4.79E-05 |
|  |  | rs373519607 | 151,943,165 | D | I | 888 | 0.8756 | 0.5531 | 0.1518 | 9.62E-05 |
| **Metadata**  ***n* = 4632** | ***NR2F6*** | rs142743962 | 17,389,183 | I | D | 4632 | 0.8788 | 0.7617 | 0.06785 | 6.02E-05 |

SNPs within the gene body are in boldface.

^1^Subdatasets of Nordic data: FIN = Finnish, MoBa = Norwegian. ^2^*NR* gene regions in GRCh37.p13 coordinates: ***RORA*** Chr15:60,780,483–61,521,502; ***ESR1*** Chr6:152,011,631–152,424,409; and ***NR2F6*** Chr19:17,342,692–17,356,744.

**Table S7. Variants with *p* < 0.0001 in HSP genes in the infant Nordic GWAS of SPTB.**

| **Dataset** | **Gene** | **Rs** | **Chr** | **Pos** | **a1** | **a2** | **Freq.** | **Eff.** | **se.** | ***p* Value** |
| --- | --- | --- | --- | --- | --- | --- | --- | --- | --- | --- |
| Finnish *n* = 817 | *HSP90AA1* | rs17541540 | 14 | 102507065 | G | C | 0.5494 | 1.5669 | 0.1145 | 8.83E-05 |
| Metadata  *n* = 1960 | *DNAJC12* | rs35717971 | 10 | 69603414 | I | D | 0.5648 | 1.34 | 0.0721 | 5.01E-05 |
|  | *CCT3* | rs41265041 | 1 | 156314440 | T | G | 0.9815 | 0.2967 | 0.3074 | 7.71E-05 |
| MoBa *n* = 1143  (**Metadata**  *n* = 1960) | *DNAJC5B* | rs144894336 | 8 | 66926558 | I | D | 0.7022 | 1.4916 | 0.0964 | 3.34E-05 |
|  |  | rs5892042 | 8 | 66918094 | D | I | 0.6986 | 1.4728 | 0.0949 | 4.48E-05 |
|  |  | rs2357569 | 8 | 66917137 | A | G | 0.6985 | 1.472 | 0.0949 | 4.58E-05 |
|  |  | rs2357568 | 8 | 66917002 | C | G | 0.6985 | 1.4719 | 0.0949 | 4.61E-05 |
|  |  | rs6990209 | 8 | 66902034 | G | A | 0.7403 | 1.4977 | 0.0993 | 4.78E-05 |
|  |  | rs10098417 | 8 | 66918380 | A | G | 0.6984 | 1.4723 | 0.0953 | 4.90E-05 |
|  |  | rs7465415 | 8 | 66920522 | T | G | 0.6983 | 1.4719 | 0.0953 | 5.02E-05 |
|  |  | rs4317552 | 8 | 66914293 | G | T | 0.6983 | 1.469 | 0.0949 | 5.03E-05 |
|  |  | rs4380924 | 8 | 66920744 | C | A | 0.6982 | 1.4717 | 0.0955 | 5.20E-05 |
|  |  | rs11997658 | 8 | 66920756 | C | T | 0.6982 | 1.4715 | 0.0955 | 5.23E-05 |
|  |  | rs7003160 | 8 | 66922377 | A | G | 0.6982 | 1.4716 | 0.0955 | 5.27E-05 |
|  |  | rs2357577 | 8 | 66923535 | A | G | 0.6982 | 1.4714 | 0.0956 | 5.35E-05 |
|  |  | rs4637811 | 8 | 66923637 | G | A | 0.6982 | 1.4713 | 0.0956 | 5.37E-05 |
|  |  | rs10097206 | 8 | 66924527 | C | G | 0.6982 | 1.4711 | 0.0956 | 5.41E-05 |
|  |  | rs10097560 | 8 | 66924814 | C | T | 0.6982 | 1.4712 | 0.0956 | 5.41E-05 |
|  |  | rs6996761 | 8 | 66911571 | T | A | 0.6982 | 1.466 | 0.0948 | 5.46E-05 |
|  |  | rs2357579 | 8 | 66926197 | T | A | 0.6982 | 1.4711 | 0.0957 | 5.46E-05 |
|  |  | rs11995702 | 8 | 66926423 | A | G | 0.6982 | 1.4712 | 0.0957 | 5.47E-05 |
|  |  | rs2357566 | 8 | 66911221 | C | T | 0.6982 | 1.4659 | 0.0948 | 5.49E-05 |
|  |  | rs6992002 | 8 | 66927237 | G | A | 0.6982 | 1.4708 | 0.0957 | 5.56E-05 |
|  |  | rs11278466 | 8 | 66927468 | I | D | 0.6982 | 1.4707 | 0.0957 | 5.60E-05 |
|  |  | rs58967538 | 8 | 66927783 | G | C | 0.6981 | 1.4708 | 0.0958 | 5.60E-05 |
|  |  | rs6472242 | 8 | 66910613 | T | G | 0.6982 | 1.4653 | 0.0948 | 5.61E-05 |
|  |  | rs28402886 | 8 | 66927891 | G | A | 0.6981 | 1.4708 | 0.0958 | 5.61E-05 |
|  |  | rs60667481 | 8 | 66928360 | G | A | 0.6981 | 1.4707 | 0.0958 | 5.65E-05 |
|  |  | rs6472241 | 8 | 66910531 | G | C | 0.6982 | 1.4651 | 0.0948 | 5.65E-05 |
|  |  | rs60020249 | 8 | 66928647 | C | T | 0.6981 | 1.4706 | 0.0958 | 5.67E-05 |
|  |  | rs28605672 | 8 | 66929194 | A | G | 0.6981 | 1.4707 | 0.0958 | 5.67E-05 |
|  |  | rs60520895 | 8 | 66930290 | C | T | 0.6981 | 1.4704 | 0.0958 | 5.74E-05 |
|  |  | rs7017618 | 8 | 66930392 | A | C | 0.6981 | 1.4703 | 0.0958 | 5.76E-05 |
|  |  | rs6983945 | 8 | 66930466 | G | C | 0.6981 | 1.4703 | 0.0958 | 5.76E-05 |
|  |  | rs12114319 | 8 | 66930916 | C | G | 0.6981 | 1.4703 | 0.0958 | 5.76E-05 |
|  |  | rs139814707 | 8 | 66908401 | C | T | 0.6984 | 1.4652 | 0.095 | 5.77E-05 |
|  |  | rs11988360 | 8 | 66931379 | G | A | 0.6981 | 1.4703 | 0.0959 | 5.80E-05 |
|  |  | rs112367238 | 8 | 66930972 | D | I | 0.698 | 1.4699 | 0.0958 | 5.83E-05 |
|  |  | rs375759138 | 8 | 66922919 | I | D | 0.6977 | 1.4687 | 0.0956 | 5.84E-05 |
|  |  | rs7812954 | 8 | 66908885 | T | C | 0.6982 | 1.4639 | 0.0949 | 5.90E-05 |
|  |  | rs77939829 | 8 | 66930988 | A | G | 0.7027 | 1.4727 | 0.0966 | 6.12E-05 |
|  |  | rs10104877 | 8 | 66932244 | C | T | 0.698 | 1.4689 | 0.0961 | 6.28E-05 |
|  |  | **rs6982577** | 8 | **66943109** | A | T | 0.6961 | 1.4721 | 0.0967 | 6.31E-05 |
|  |  | **rs7002734** | 8 | **66942971** | T | A | 0.6961 | 1.4717 | 0.0966 | 6.37E-05 |
|  |  | **rs2884235** | 8 | **66934767** | T | C | 0.6979 | 1.4683 | 0.0961 | 6.40E-05 |
|  |  | rs4487748 | 8 | 66910028 | G | A | 0.7134 | 1.489 | 0.0996 | 6.43E-05 |
|  |  | **rs7006789** | 8 | **66935288** | G | A | 0.6979 | 1.4681 | 0.0961 | 6.47E-05 |
|  |  | **rs10092435** | 8 | **66936177** | A | G | 0.6979 | 1.4681 | 0.0961 | 6.49E-05 |
|  |  | **rs2357657** | 8 | **66936490** | C | A | 0.6979 | 1.468 | 0.0961 | 6.50E-05 |
|  |  | **rs7010679** | 8 | **66935691** | A | T | 0.6979 | 1.4679 | 0.0961 | 6.50E-05 |
|  |  | **rs62507391** | 8 | **66936742** | C | T | 0.6979 | 1.4678 | 0.0962 | 6.57E-05 |
|  |  | rs9694378 | 8 | 66905674 | T | C | 0.6982 | 1.4608 | 0.095 | 6.57E-05 |
|  |  | **rs55818650** | 8 | **66939804** | D | I | 0.6959 | 1.469 | 0.0965 | 6.74E-05 |
|  |  | rs11386093 | 8 | 66905093 | D | I | 0.6986 | 1.4604 | 0.0951 | 6.83E-05 |
|  |  | rs62506641 | 8 | 66903691 | C | T | 0.6981 | 1.4589 | 0.095 | 6.99E-05 |
|  |  | rs112287108 | 8 | 66903690 | D | I | 0.6981 | 1.4588 | 0.095 | 7.00E-05 |
|  |  | rs35992327 | 8 | 66921100 | D | I | 0.7141 | 1.4722 | 0.0975 | 7.34E-05 |
|  |  | **rs4262311** | 8 | **66943667** | T | A | 0.6944 | 1.4654 | 0.0965 | 7.49E-05 |
|  |  | rs7010113 | 8 | 66901991 | T | C | 0.6982 | 1.4562 | 0.095 | 7.58E-05 |
|  |  | rs6985149 | 8 | 66901401 | A | G | 0.6973 | 1.4561 | 0.095 | 7.66E-05 |
|  |  | rs9918852 | 8 | 66901842 | A | G | 0.6982 | 1.4557 | 0.095 | 7.69E-05 |
|  |  | rs6989850 | 8 | 66901776 | G | T | 0.6982 | 1.4555 | 0.095 | 7.72E-05 |
|  |  | **rs2357666** | 8 | **66954615** | C | A | 0.7188 | 1.4747 | 0.099 | 8.66E-05 |
|  |  | rs4355757 | 8 | 66920724 | C | T | 0.7053 | 1.4563 | 0.0959 | 8.85E-05 |
|  |  | rs16932411 | 8 | 66922465 | A | G | 0.7052 | 1.4562 | 0.096 | 8.99E-05 |
|  |  | rs55864718 | 8 | 66924833 | A | G | 0.7052 | 1.4555 | 0.096 | 9.30E-05 |
|  |  | rs11995587 | 8 | 66926045 | A | G | 0.7052 | 1.4554 | 0.0961 | 9.39E-05 |
|  |  | rs11995704 | 8 | 66926428 | A | G | 0.7052 | 1.4552 | 0.0961 | 9.47E-05 |
|  |  | **rs2357663** | 8 | **66947940** | G | A | 0.7112 | 1.4636 | 0.0978 | 9.86E-05 |

*HSP* gene regions in GRCh37.p13 coordinates: ***HSP90AA1*** Chr14:102,547,075–102,606,086; ***DNAJC12*** Chr10:69,556,427–69,597,937; ***CCT3*** Chr1:156,278,752–156,308,206; ***DNAJC5B*** Chr8:66,933,791–67,012,755.

**Table S8. *HSPA12B* variants with *p* < 0.0001 in the infant Northern Finnish GWAS data.**

| **SNP** | **Position** | **AlleleA** | **AlleleB** | **All_maf** | **Cases_maf** | **Controls_maf** | **all_OR** | ***p* Value** |
| --- | --- | --- | --- | --- | --- | --- | --- | --- |
| **rs58505239** | **3731139** | C | T | 0.205 | 0.264 | 0.170 | 1.748 | 8.66E-06 |
| **rs3827077** | **3721456** | C | T | 0.084 | 0.125 | 0.059 | 2.252 | 2.22E-05 |
| **rs6139190** | **3723581** | C | T | 0.084 | 0.124 | 0.060 | 2.223 | 3.34E-05 |
| **rs79423311** | **3723868** | T | TCA | 0.083 | 0.124 | 0.060 | 2.219 | 3.48E-05 |
| **rs373564953** | **3724401** | TACAAA | T | 0.083 | 0.124 | 0.060 | 2.221 | 3.54E-05 |
| **rs78012956** | **3724123** | C | T | 0.083 | 0.124 | 0.060 | 2.219 | 3.54E-05 |
| **rs6107337** | **3717101** | G | T | 0.097 | 0.138 | 0.073 | 2.041 | 4.32E-05 |
| **rs6139191** | **3724708** | A | G | 0.086 | 0.126 | 0.062 | 2.169 | 4.69E-05 |
| **rs6116008** | **3715219** | T | C | 0.098 | 0.138 | 0.074 | 2.018 | 4.98E-05 |
| **rs910652** | **3727970** | T | C | 0.202 | 0.254 | 0.171 | 1.644 | 8.48E-05 |

All listed variants were located within the gene.

*HSPA12B* in GRCh37.p13 coordinates: Chr20:3,713,317–3,733,758.
